# Supplementary material for: A Phenome-Wide Association Study (PheWAS) of COVID-19 Outcomes by Race Using the Electronic Health Records Data in Michigan Medicine
Source: J Clin Med. 2021 Mar 25;10(7):1351. doi: 10.3390/jcm10071351 (PMC8037108; doi:10.3390/jcm10071351)
Supplement: Supplementary file 1 [file jcm-10-01351-s001.pdf]

Supplementary Material

A Phenome-Wide Association Study (PheWAS) of COVID-19 Outcomes by Race using the Electronic Health Records Data in Michigan Medicine

Control selection for susceptibility PheWAS. Controls were used in the susceptibility models and not in the prognosis models. We created two sets of alive control samples from the MM patient database to compare and contrast the testing positive, one “unmatched control sample” consists of 29,383 randomly drawn patients and another 47,510 “matched control sample” using 1:3 frequency-matching on race, ethnicity, sex and age (above/below 50) based on a previous cohort of about 16,000 tested individuals. We used unmatched controls in the analysis with the full cohort (including all races) and matched controls in the race-stratified analysis. Matched controls were used in the race-stratified models for COVID-19 susceptibility as the proportion of non-Hispanic Whites (White) and non-Hispanic Black/African American (Black) in unmatched controls are not comparable to the stratified population under study.

There were 53,853 patients who were either tested for or diagnosed with COVID-19, 29,383 unmatched controls ($n_{unmatched}$), and 47,510 matched controls ($n_{matched}$) eligible for inclusion in this study. Of the 130,746 individuals eligible for inclusion, our study population comprised 123,344 individuals (n_{tested}=47,862 [$n_{positive}$=2,133]; $n_{unmatched}$=28,505; $n_{matched}$= 46,977) who had available International Classification of Disease (ICD; ninth and tenth editions) code data after applying the 14-day-prior to testing restriction to the EHR. Furthermore, a total of 1,813 qualified ICD-code-based phenotypes, referred to as PheWAS codes, were initially screened of which 1,363 had at least 10 occurrences in our COVID-19 positive cohort and were included in the analysis.

Full cohort susceptibility. For susceptibility, when comparing the positives and the unmatched controls, 929 traits were identified as significant after applying Bonferroni correction, including 101 genitourinary, 97 circulatory system, 91 endocrine/metabolic, and 89 digestive PheWAS codes. This suggests that patients who tested positive were enriched for pre-existing conditions compared to the control population. As illustrated in **Figure S4A**, we found strong and positive associations with various comorbidities and COVID-19 positive diagnosis (e.g., shortness of breath [P=1.00x10^{-127}], cough [P=1.00x10^{-119}], pneumonia [P=1.00x10^{-108}], nonspecific chest pain [P=7.94x10^{-95}], vitamin D deficiency [P=1.26x10^{-70}] and anxiety disorders [P=5.01x10^{-53}]). Overall, the findings were consistent

with previously identified COVID-19 risk factors (e.g., obesity [$P=1.26\times10^{-95}$] and diabetes mellitus [$P=3.16\times10^{-64}$] were associated with higher risk of being test positive) (**Table S3**).[19] In contrast, the comparison between those who tested positive for COVID-19 and those who tested negative leads to counterintuitive findings (all 137 significant traits under Bonferroni correction showed protective effect, such as cataract [odds ratio [OR]=0.45, $P=6.31\times10^{-7}$] and asthma [OR=0.68, $P=3.98\times10^{-18}$]) contradicting findings in other COVID-19 studies.[20,21] However, this can likely be explained by the applied eligibility criteria, e.g., a larger proportion of COVID-19-positive individuals became eligible due to symptom manifestation while COVID-19-negative individuals were tested due to Michigan's priority testing criteria[22] (without symptoms). This amplifies the need for choosing an appropriate control group.

Race-stratified susceptibility analysis. As shown in **Figure S4B**, we identified 871 traits in Whites, including 122 circulatory system, 120 digestive, 119 genitourinary, and 107 endocrine/metabolic diseases that reached significance under Bonferroni correction. Examples of the top traits include cough ($P=3.98\times10^{-84}$), nonspecific chest pain ($P=3.16\times10^{-68}$), malaise and fatigue ($P=2.00\times10^{-64}$), abnormal glucose ($P=1.58\times10^{-58}$), and pain in joint ($P=1.26\times10^{-56}$). As illustrated in **Figure S4C**, we observed 584 significant traits in Blacks under Bonferroni correction, including 118 circulatory system, 110 genitourinary, 102 endocrine/metabolic, 99 digestive and 68 musculoskeletal diseases, where some of the top traits includes nonspecific chest pain ($P=2.51\times10^{-37}$), electrolyte imbalance ($P=1.00\times10^{-36}$), acute renal failure ($P=1.26\times10^{-35}$) and cardiac dysrhythmias ($P=2.00\times10^{-33}$).

Figure S1. Flowchart describing the sample sizes of the overall and race-specific cohorts by progression of COVID-19 outcomes.

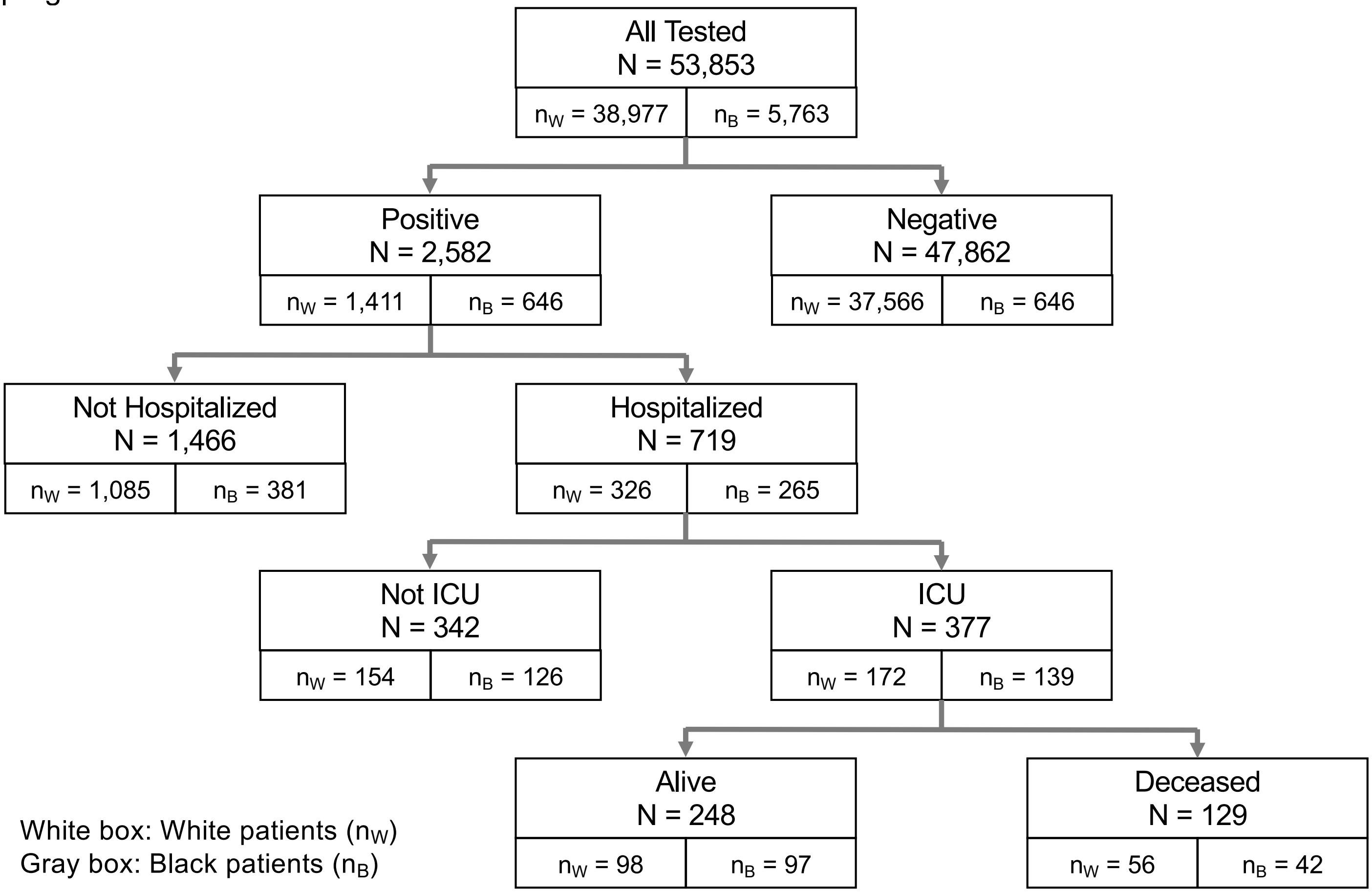

Figure S2. Forest plots of traits associated with poorer prognosis reaching phenome-wide significance in the overall cohort. *A. Hospitalization. B. ICU admission/mortality. Odds ratios and 95% confidence intervals are shown for each trait whose PheWAS code is given in parentheses. Plots show parent codes only. Child codes found in Figure S3.*

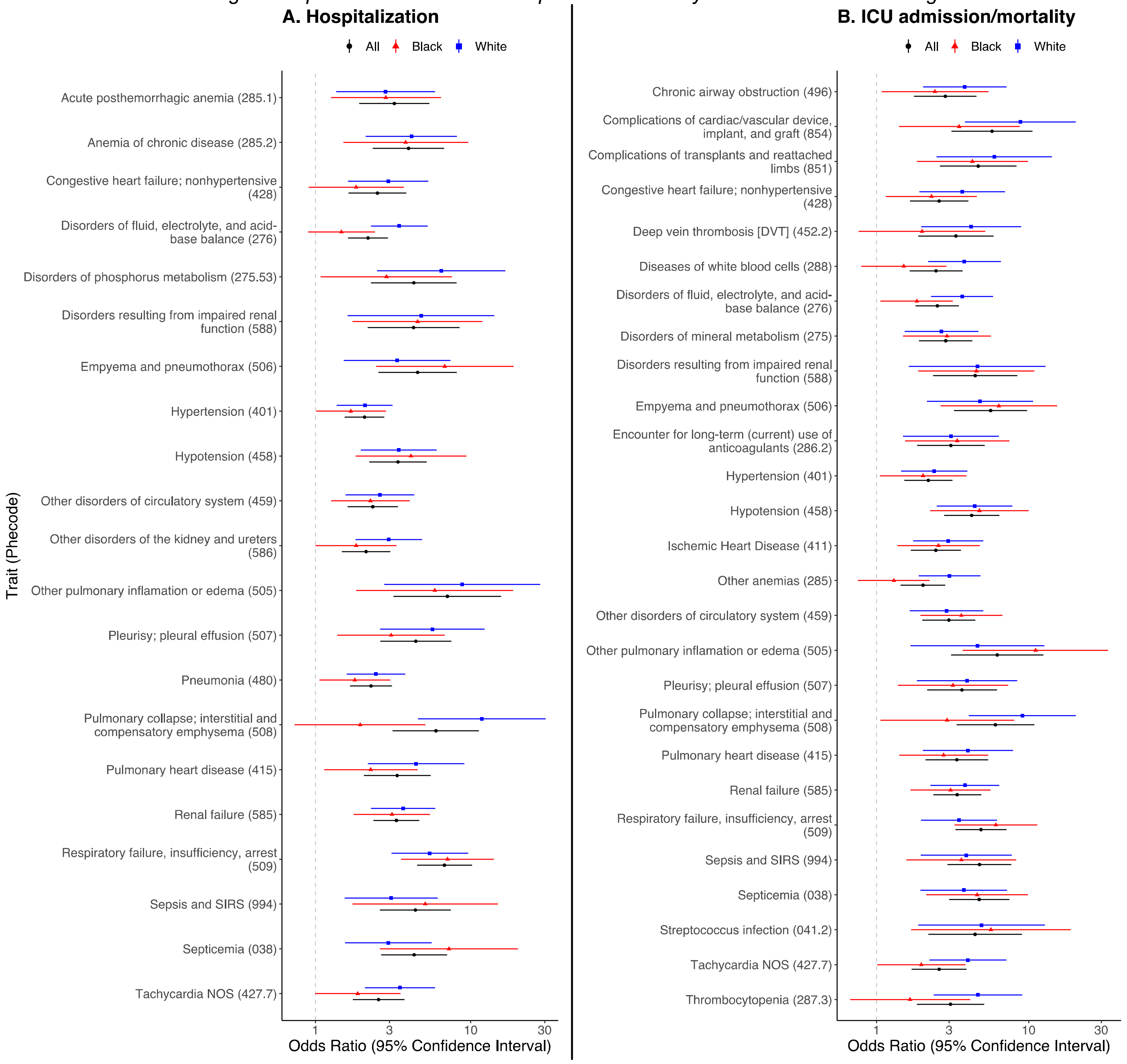

Figure S3. Forest plots of traits associated with poorer prognosis in overall cohort. *A. Hospitalization. B. ICU admission/mortality. Odds ratios and 95% confidence intervals are shown for each trait whose PheWAS code is given in parentheses. Plots show child codes only. Parent codes found in Figure S2.*

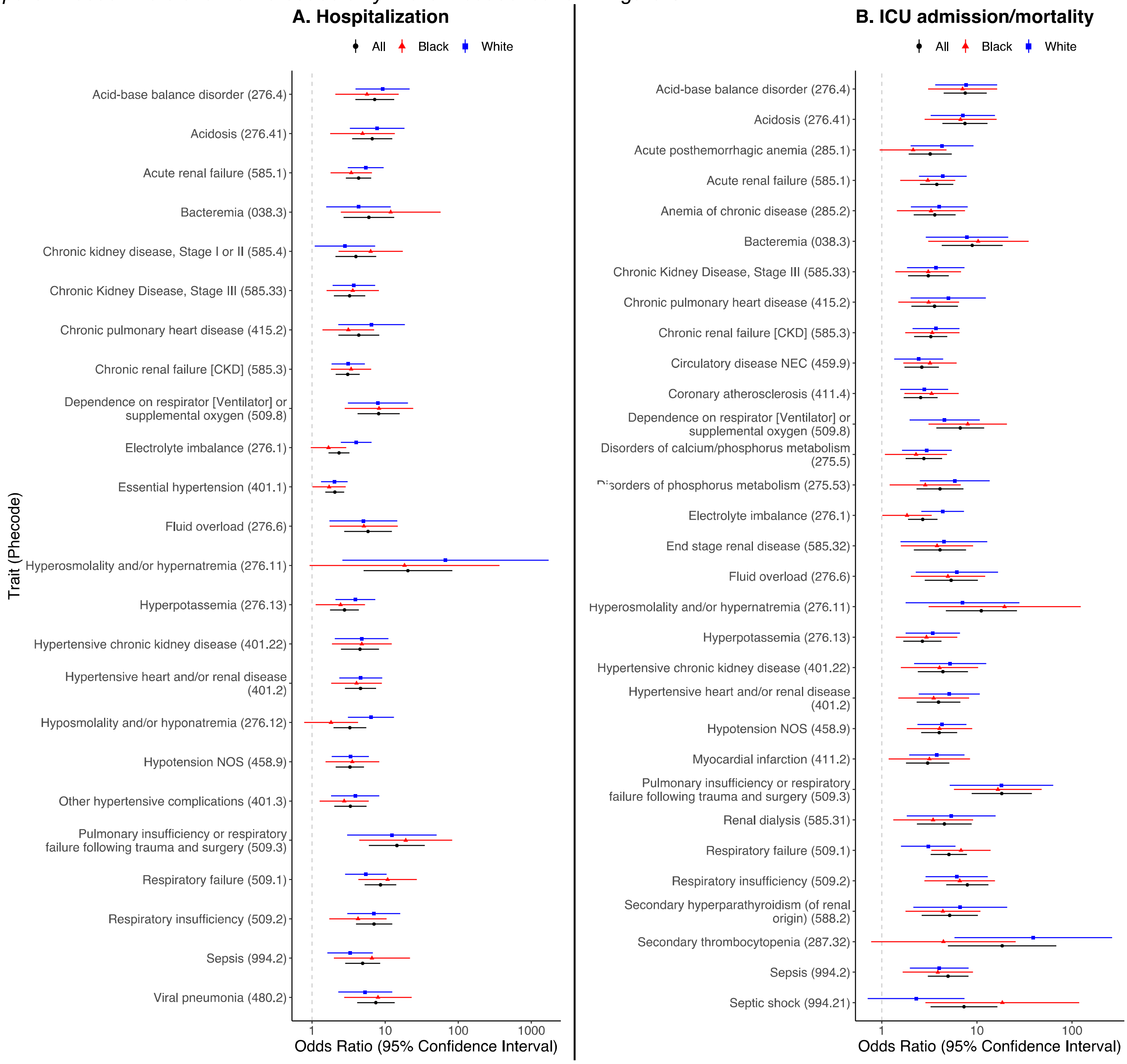

Figure S4. Forest plot showing nominally significant effect differences comparing Whites and Blacks with respect to COVID-19-related hospitalization.

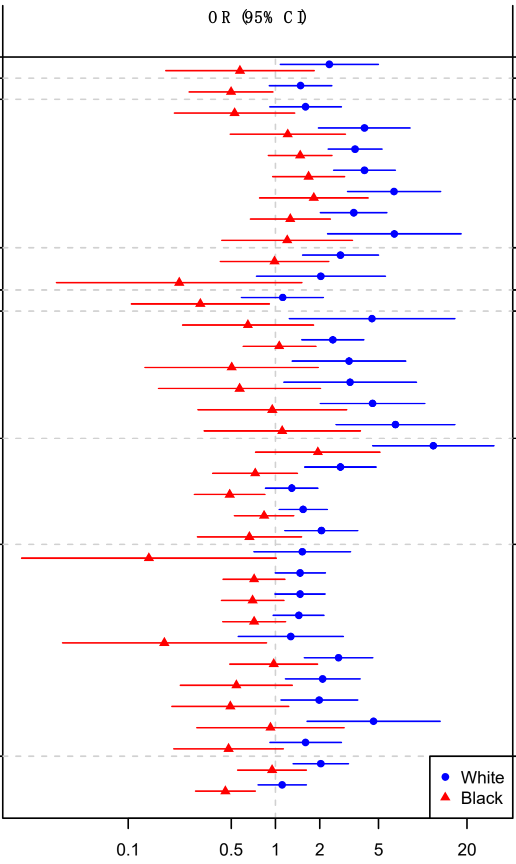

Predictor	Phecode	White OR	White 95% CI (L, U)		Black OR	Black 95% CI (L, U)		P White	P Black	P Diff.
Bacterial enteritis	008.5	2.32	1.08	4.98	0.57	0.18	1.83	0.031	0.35	0.048
Benign neoplasm of other parts o...	211	1.48	0.91	2.41	0.50	0.26	0.96	0.12	0.038	0.0090
Other disorders of thyroid	246	1.60	0.92	2.80	0.53	0.21	1.35	0.099	0.18	0.047
Protein−calorie malnutrition	260	4.02	1.96	8.21	1.21	0.49	2.98	0.00014	0.67	0.041
Disorders of fluid, electrolyte,...	276	3.47	2.28	5.29	1.47	0.89	2.42	6.2e−09	0.13	0.0093
Electrolyte imbalance	276.1	4.02	2.48	6.51	1.68	0.96	2.94	1.5e−08	0.070	0.021
Hyposmolality and/or hyponatremia	276.12	6.38	3.09	13.2	1.82	0.78	4.25	5.7e−07	0.17	0.028
Hypovolemia	276.5	3.40	2.02	5.71	1.26	0.68	2.36	3.9e−06	0.46	0.017
Immunity deficiency	279.1	6.41	2.26	18.2	1.20	0.43	3.33	0.00047	0.73	0.024
Alteration of consciousness	291.8	2.76	1.53	5.00	0.98	0.42	2.30	0.00078	0.97	0.050
Posttraumatic stress disorder	300.9	2.03	0.74	5.57	0.22	0.03	1.51	0.17	0.12	0.046
Impacted cerumen	380.4	1.12	0.59	2.11	0.31	0.10	0.91	0.74	0.033	0.045
Pericarditis	420.2	4.52	1.24	16.5	0.65	0.23	1.81	0.022	0.41	0.021
Abnormal function study of cardi...	429.2	2.45	1.51	3.98	1.06	0.60	1.88	0.00029	0.83	0.028
Occlusion and stenosis of precer...	433.1	3.16	1.30	7.65	0.50	0.13	1.95	0.011	0.32	0.026
Other aneurysm	442	3.21	1.14	9.05	0.57	0.16	2.02	0.027	0.39	0.038
Other specified peripheral vascu...	443.8	4.56	2.02	10.3	0.95	0.30	3.04	0.00026	0.93	0.030
Peripheral vascular disease, uns...	443.9	6.53	2.58	16.5	1.11	0.33	3.77	7.5e−05	0.86	0.023
Pulmonary collapse; interstitial...	508	11.8	4.57	30.4	1.94	0.73	5.11	3.4e−07	0.18	0.0090
Other diseases of lung	510	2.76	1.58	4.82	0.73	0.38	1.41	0.00034	0.35	0.0024
Cough	512.8	1.29	0.85	1.94	0.49	0.28	0.85	0.23	0.011	0.0057
Respiratory abnormalities	513	1.54	1.06	2.25	0.84	0.53	1.33	0.024	0.45	0.044
Abnormal findings examination of...	514	2.05	1.16	3.61	0.66	0.30	1.50	0.013	0.32	0.026
Diseases of the jaws	526	1.52	0.71	3.23	0.14	0.02	1.01	0.28	0.051	0.027
Diseases of esophagus	530	1.47	1.00	2.18	0.72	0.44	1.16	0.051	0.17	0.023
Esophagitis, GERD and related di...	530.1	1.47	0.99	2.17	0.70	0.43	1.14	0.055	0.15	0.020
GERD	530.11	1.44	0.97	2.13	0.72	0.44	1.17	0.074	0.18	0.030
Heartburn	530.9	1.27	0.56	2.89	0.18	0.04	0.87	0.57	0.033	0.030
Dysphagia	532	2.68	1.57	4.57	0.97	0.49	1.93	0.00028	0.94	0.022
Chronic liver disease and cirrhosis	571	2.09	1.17	3.75	0.54	0.23	1.30	0.013	0.17	0.012
Other chronic nonalcoholic liver...	571.5	1.98	1.09	3.61	0.49	0.20	1.23	0.026	0.13	0.013
Ascites (non malignant)	572	4.63	1.64	13.1	0.92	0.29	2.92	0.0038	0.89	0.042
Other disorders of liver	573	1.60	0.92	2.80	0.48	0.20	1.13	0.098	0.094	0.021
Fever of unknown origin	783	2.03	1.32	3.13	0.95	0.55	1.62	0.0014	0.84	0.031
Abdominal pain	785	1.11	0.76	1.62	0.46	0.29	0.73	0.58	0.0010	0.0037

Figure S5. Forest plot showing nominally significant effect differences comparing Whites and Blacks with respect to COVID-19-related ICU admission.

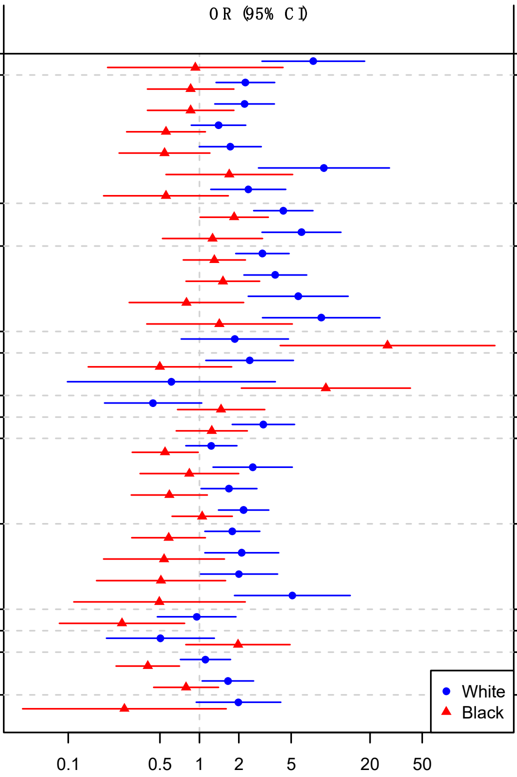

Predictor	Phecode	White OR	White 95% CI (L, U)		Black OR	Black 95% CI (L, U)		P White	P Black	P Diff.
Staphylococcus infections	041.1	7.38	3.00	18.2	0.93	0.20	4.34	1.4e−05	0.93	0.023
Cancer, suspected or other	195	2.24	1.34	3.75	0.86	0.40	1.83	0.0021	0.69	0.040
Malignant neoplasm, other	195.1	2.21	1.31	3.73	0.86	0.40	1.83	0.0028	0.69	0.043
Benign neoplasm of colon	208	1.40	0.87	2.25	0.56	0.28	1.11	0.17	0.094	0.031
Benign neoplasm of other parts o...	211	1.72	1.00	2.96	0.54	0.24	1.20	0.052	0.13	0.019
Benign neoplasm of respiratory a...	212	8.89	2.82	28.1	1.69	0.56	5.14	0.00019	0.35	0.042
Other benign neoplasm of connect...	215	2.36	1.22	4.56	0.56	0.19	1.66	0.011	0.29	0.027
Electrolyte imbalance	276.1	4.36	2.60	7.32	1.84	1.01	3.35	2.6e−08	0.045	0.034
Hyposmolality and/or hyponatremia	276.12	6.00	3.00	12.0	1.26	0.52	3.03	4.1e−07	0.61	0.0063
Other anemias	285	3.02	1.89	4.82	1.30	0.75	2.24	3.6e−06	0.35	0.021
Diseases of white blood cells	288	3.78	2.18	6.57	1.51	0.79	2.88	2.2e−06	0.21	0.034
Decreased white blood cell count	288.1	5.67	2.36	13.6	0.80	0.29	2.17	1e−04	0.66	0.0038
Neutropenia	288.11	8.50	3.03	23.8	1.42	0.40	5.11	4.8e−05	0.59	0.033
Delirium due to conditions class...	290.2	1.86	0.73	4.76	27.2	4.13	179	0.20	0.00059	0.013
Hypersomnia	327.1	2.42	1.12	5.21	0.50	0.14	1.76	0.024	0.28	0.036
Chronic pain syndrome	355.1	0.61	0.10	3.79	9.20	2.09	40.6	0.60	0.0034	0.024
Disorders of lacrimal system	375	0.44	0.19	1.04	1.46	0.68	3.15	0.060	0.33	0.040
Abnormal function study of cardi...	429.2	3.07	1.78	5.31	1.24	0.66	2.32	5.8e−05	0.50	0.033
Acute upper respiratory infectio...	465	1.23	0.79	1.93	0.55	0.31	0.98	0.36	0.041	0.029
Painful respiration	512.2	2.55	1.27	5.11	0.84	0.35	1.99	0.0085	0.69	0.050
Cough	512.8	1.68	1.03	2.74	0.59	0.30	1.15	0.038	0.12	0.013
Respiratory abnormalities	513	2.17	1.40	3.38	1.05	0.62	1.78	0.00056	0.86	0.038
Functional digestive disorders	564	1.78	1.10	2.89	0.58	0.30	1.11	0.018	0.10	0.0065
Chronic liver disease and cirrhosis	571	2.10	1.10	4.02	0.54	0.19	1.55	0.025	0.25	0.031
Other chronic nonalcoholic liver...	571.5	2.00	1.02	3.94	0.51	0.16	1.58	0.045	0.24	0.042
Ascites (non malignant)	572	5.11	1.85	14.1	0.50	0.11	2.23	0.0016	0.36	0.012
Abnormal findings on mammogram o..	611	0.95	0.48	1.90	0.26	0.09	0.77	0.89	0.015	0.047
Degeneration of intervertebral disc	722.6	0.50	0.20	1.30	1.97	0.79	4.91	0.16	0.15	0.043
Abdominal pain	785	1.11	0.72	1.73	0.40	0.23	0.70	0.64	0.0014	0.0051
Malaise and fatigue	798	1.65	1.05	2.59	0.79	0.45	1.40	0.029	0.42	0.047
Fracture of ankle and foot	801	1.98	0.95	4.17	0.27	0.04	1.60	0.070	0.15	0.042

Figure S6. Forest plot showing nominally significant effect differences comparing Whites and Blacks with respect to COVID-19-related death.

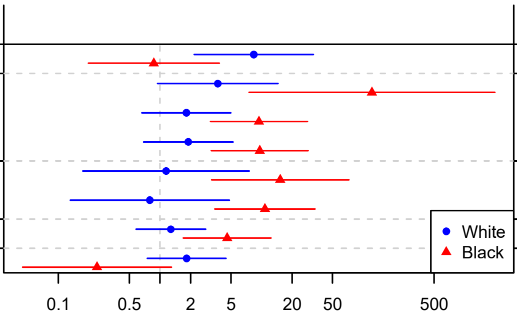

		OR	95% CI (L,	U)	OR	95% CI (L,	U)	White	Black	Diff.
Decreased white blood cell count	288.1	8.38	2.17	32.4	0.87	0.20	3.84	0.0021	0.85	0.027
First degree AV block	426.21	3.71	0.95	14.5	122	7.53	1980	0.060	0.00072	0.027
Atrial fibrillation and flutter	427.2	1.82	0.66	4.99	9.42	3.14	28.3	0.24	6.3e−05	0.031
Atrial fibrillation	427.21	1.90	0.69	5.23	9.60	3.20	28.8	0.22	5.6e−05	0.034
Pulmonary congestion and hypostasis	503	1.15	0.17	7.57	15.3	3.22	72.4	0.89	0.00059	0.038
Empyema and pneumothorax	506	0.79	0.13	4.82	10.8	3.47	33.6	0.80	4e−05	0.016
Disorder of skin and subcutaneou...	689	1.28	0.58	2.82	4.58	1.70	12.4	0.54	0.0027	0.049
Cervicalgia	761	1.83	0.75	4.47	0.24	0.04	1.30	0.18	0.098	0.037

Figure S7. Manhattan plot showing the phenome-wide association between disease codes and testing positive for COVID-19. *Models are adjusted for age, sex, race (full cohort only), and four census tract-level socioeconomic indicators: proportion with less than high school education, proportion unemployed, proportion with annual income below the federal poverty level, and population density (persons per mile^2). The x-axis are individual disease codes, color-coded by their corresponding disease category as described in the legend. The y-axis represents the -log$_{10}$ transformed p-value of the association. The dashed, horizontal lines represent the p = 0.05 (in orange) and the Bonferroni corrected p-value (0.05 / number of tests; in red). Each point is represented by either an upward triangle indicating a positive association or a downward triangle indicating a negative association.*

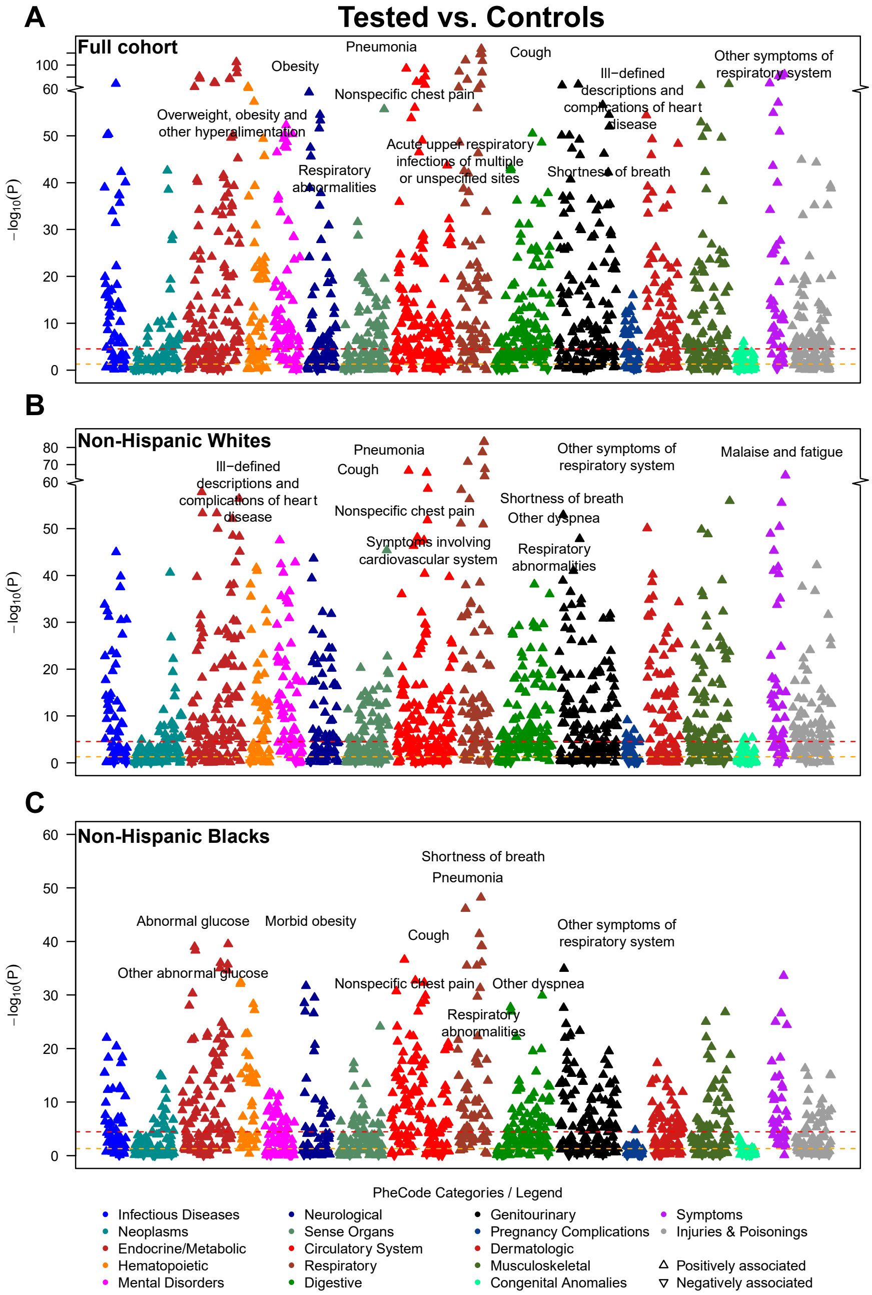

Table S1A. Definition and Sources of All Variables

	Definition	Sources
Age	Age of patient as of the data pull: 4/22/2020	Electronic Health Record (EPIC)
Male	Gender of patient as reported.	Electronic Health Record (EPIC)
BMI	Excluded entries if (1) age at BMI measurement was missing or below 18 years, (2) height and/or weight were missing, (3) height measurements were below 69 cm or above 234 cm, (4) weight was above 400 kg, (5) BMI deviated more than one unit from BMI calculated from height and weight (BMI = weight [in kg] / height [in m]^2). Outliers for multiple values per person were defined as values that exceeded the median BMI +/- 3 x the median absolute deviation (MAD). Final BMI values was calculated as the median BMI of the remaining entries.	Derived from the Electronic Health Record
**Race/Ethnicity**		
White	If race was reported as "Caucasian" and ethnicity as "Hispanic or Latino"	Patient Reported - Derived from the Electronic Health Record
Black	If race was reported as "African American" and ethnicity as "Hispanic or Latino"	
Other / Known Ethnicity	If race was not reported as "African American" or "Caucasian"' and ethnicity was reported as "Non-Hispanic or Latino" or "Hispanic or Latino"	
Other / Unknown Ethnicity	If race and/or ethnicity were missing	
**SES**	Data defined by US census tract (based on residential address available in each patient's EHR) for the year 2010 from the US Census and the American Community Survey (ACS).	The boundaries for the census tracts were normalized by 2010 tract boundaries using the Longitudinal Tract Data Base (Logan, Xu, and Stults, 2014).
Less than HS Education	Proportion of the Neighborhood Census Tract that has less than a High School Education	
Unemployed	Proportion of the Neighborhood Census Tract age 16+ in the civilian labor force who are unemployed in 2010	
Annual Income Below FPL	Proportion of the Neighborhood Census Tract with annual income below the federal poverty level in 2010	
Persons Per Mile^2	Population Density of the neighborhood that the patient lives in.	
**COVID-19 Outcomes**		
COVID-19 Tested	Patients who were tested for COVID-19 at the time of data pull.	Derived from RDW's COVID Registry
COVID-19 Positive	Patients who tested positive at least once for COVID-19	
COVID-19 Negative	Patients who always tested negative for COVID-19	
Not-Hospitalized	Patients in the positive COVID cohort who have no inpatient stays after 3/5/2020	
Hospitalized	Patients in the positive COVID cohort who checked in as an inpatient after 3/5/2020 at least once.	
ICU	Patients in the positive COVID cohort who checked into the ICU during their inpatient stay after 3/5/2020.	
Deceased	Patients in the cohort who have died based on the Electronic Health Record.	Electronic Health Record (EPIC)
**Control Groups**		
Unmatched Control	Randomly picked cohort of patients who are not part of the COVID-19 cohort (Tested, Positive, Negative), who are alive and who have had an encounter in MM (Inpatient, Outpatient or Emergency) since 2012-04-23	Derived from the Electronic Health Record
Matched Control	Randomly picked cohort of patients matched by Age (<=50, >50), Race/Ethnicity, Gender to be 3 times the COVID cohort for each group. Find attached table for frequency matching.	

Abbreviations: Black, non-Hispanic Black/African American; BMI, body mass index; MM, Michigan Medicine; SES, social economics status; HS, high school; FPL, federal poverty level; White, non-Hispanic White.

Table S1B. Analysis Setup

Full Cohort	Main Analysis						Sensitivity Analysis				
	Tested (1) vs. Controls (0)	Positive (1) vs. Controls (0)	Positive (1) vs. Negative (0)	Hospitalized (1) vs. Not (0)	ICU (1) vs. Not (0)	Deceased (1) vs. Alive (0)	Positive (1) vs. Controls (0)	Positive (1) vs. Negative (0)	Hospitalized (1) vs. Not (0)	ICU (1) vs. Not (0)	Deceased (1) vs. Alive (0)
Not hospitalized	1	1	1	0	0	0	1	1	0	0	0
Not hospitalized / Deceased	1	1	1	1	1	1	1	1	1	1	1
Still hospitalized	1	1	1	1	0	0	NA	NA	NA	NA	NA
Hospitalized / Discharged	1	1	1	1	0	0	1	1	1	0	0
Hospitalized / Deceased	1	1	1	1	1	1	1	1	1	1	1
Still in ICU	1	1	1	1	1	0	NA	NA	NA	NA	NA
ICU / Discharged	1	1	1	1	1	0	1	1	1	1	0
ICU / Deceased	1	1	1	1	1	1	1	1	1	1	1
Matched Controls	NA	NA	NA	NA	NA	NA	NA	NA	NA	NA	NA
Unmatched Controls	0	0	NA	NA	NA	NA	0	NA	NA	NA	NA
Tested Negative	1	NA	0	NA	NA	NA	NA	0	NA	NA	NA
Adjustment 1	Age, Gender, Race/Ethnicity, Persons Per Mile^2			Age, Gender, Race/Ethnicity			Age, Gender, Race/Ethnicity, Persons Per Mile^2		Age, Gender, Race/Ethnicity		
Adjustment 2	Adjustment 1 + <HS education, Unemployed, Annual Income Below FPL										
Adjustment 3	Adjustment 2 + Comorbidity Score										

Race/ethnicity Stratified	Positive (1) vs. Controls (0)	Hospitalized (1) vs. Not (0)	ICU (1) vs. Not (0)	Dead (1) vs Alive (0)
Not hospitalized	1	0	0	0
Not hospitalized / Deceased	1	1	1	1
Still hospitalized	1	1	0	0
Hospitalized / Discharged	1	1	0	0
Hospitalized / Deceased	1	1	1	1
Still in ICU	1	1	1	0
ICU / Discharged	1	1	1	0
ICU / Deceased	1	1	1	1
Matched Controls	0	NA	NA	NA
Unmatched Controls	NA	NA	NA	NA
Tested Negative	NA	NA	NA	NA
Adjustment 1	Persons Per Mile^2	Age, Gender		
Adjustment 2	Adjustment 1 + <HS education, Unemployed, Annual Income Below FPL			
Adjustment 3	Adjustment 2 + Comorbidity Score			

Abbreviations: Black, non-Hispanic Black/African American; BMI, body mass index; MM, Michigan Medicine; SES, social economics status; HS, high school; FPL, federal poverty level; White, non-Hispanic White.

Table S2. Descriptive cohort characteristics stratified by race

Non-Hispanic Whites

Variable	Statistics	Tested	Tested Negative	Tested Positive	Hospitalized	ICU	Deceased	Unmatched Controls
n		38977	37566	1411	326	172	56	18529
Age	mean (SD)	46.1 (23.3)	46 (23.4)	47 (20.3)	59.9 (18.1)	59.4 (18.5)	72.2 (15.7)	43 (24.5)
	median (IQR)	49 (37)	49 (37)	49 (34)	63 (22)	63 (22)	77 (18.8)	43 (42)
AgeCategory	n (%)							
[0,18)		5020 (12.9)	4951 (13.2)	69 (4.9)	7 (2.1)	5 (2.9)	0 (0)	3450 (18.6)
[18,35)		8304 (21.3)	7929 (21.1)	375 (26.6)	31 (9.5)	17 (9.9)	3 (5.4)	4232 (22.8)
[35,50)		6345 (16.3)	6058 (16.1)	287 (20.3)	40 (12.3)	20 (11.6)	3 (5.4)	2816 (15.2)
[50,65)		9063 (23.3)	8702 (23.2)	361 (25.6)	100 (30.7)	51 (29.7)	8 (14.3)	3560 (19.2)
[65,80)		8177 (21)	7928 (21.1)	249 (17.6)	105 (32.2)	61 (35.5)	18 (32.1)	3320 (17.9)
[80,100)		2068 (5.3)	1998 (5.3)	70 (5)	43 (13.2)	18 (10.5)	24 (42.9)	1151 (6.2)
Gender	(Male; %)	17327 (44.5)	16672 (44.4)	655 (46.4)	196 (60.1)	114 (66.3)	38 (67.9)	8606 (46.5)
Primary Care in MM	(Yes; %)	23813 (61.1)	22935 (61.1)	878 (62.2)	139 (42.6)	81 (47.1)	22 (39.3)	2939 (15.9)
BMI	mean (SD)	29 (7.3)	29 (7.3)	29.6 (7.4)	31.2 (8.2)	30.5 (8.4)	30 (6.9)	28.5 (7.3)
BMIcategory	n (%)							
[0,18.5)		571 (1.8)	559 (1.8)	12 (1)	4 (1.3)	4 (2.4)	1 (1.9)	243 (2)
[18.5,25)		9718 (29.8)	9366 (29.9)	352 (28.4)	57 (18.3)	37 (22.4)	8 (14.8)	3826 (32)
[25,30)		10187 (31.3)	9799 (31.3)	388 (31.3)	103 (33)	56 (33.9)	23 (42.6)	3781 (31.7)
[30,200)		12085 (37.1)	11598 (37)	487 (39.3)	148 (47.4)	68 (41.2)	22 (40.7)	4090 (34.3)
Smoker	(Ever; %)	14197 (38.3)	13773 (38.5)	424 (33.2)	134 (45.4)	73 (48.3)	22 (55)	5378 (33.3)
SmokingStatus	n (%)							
Never		22832 (61.7)	21978 (61.5)	854 (66.8)	161 (54.6)	78 (51.7)	18 (45)	10763 (66.7)
Past		10999 (29.7)	10630 (29.7)	369 (28.9)	120 (40.7)	66 (43.7)	21 (52.5)	3707 (23)
Current		3198 (8.6)	3143 (8.8)	55 (4.3)	14 (4.7)	7 (4.6)	1 (2.5)	1671 (10.4)
Drinker	(Yes; %)	20623 (70.9)	19894 (70.9)	729 (69.7)	151 (69.3)	78 (66.7)	22 (71)	6718 (57.3)
NDI	mean (SD)	0.09 (0.06)	0.09 (0.06)	0.09 (0.06)	0.09 (0.06)	0.1 (0.06)	0.1 (0.05)	0.1 (0.06)
PersonsPerSquareMile	mean (SD)	2106.4 (2263.9)	2094.9 (2263.5)	2405.7 (2252.8)	2711.6 (2228.1)	2895.7 (2343.9)	2880.7 (1950.8)	2057.5 (2316.2)
Respiratory Diseases	(Yes; %)	26239 (72.8)	25253 (72.7)	986 (77.5)	204 (79.7)	113 (85.6)	37 (92.5)	8645 (48)
Circulatory Diseases	(Yes; %)	24802 (68.9)	23939 (68.9)	863 (67.8)	219 (85.5)	115 (87.1)	37 (92.5)	8372 (46.4)
Any Cancer	(Yes; %)	11276 (31.3)	10957 (31.5)	319 (25.1)	100 (39.1)	58 (43.9)	23 (57.5)	3540 (19.6)
Type 2 Diabetes	(Yes; %)	5573 (15.5)	5368 (15.4)	205 (16.1)	85 (33.2)	47 (35.6)	23 (57.5)	1579 (8.8)
Kidney Diseases	(Yes; %)	5360 (14.9)	5193 (14.9)	167 (13.1)	89 (34.8)	54 (40.9)	24 (60)	919 (5.1)
Liver Diseases	(Yes; %)	3478 (9.7)	3372 (9.7)	106 (8.3)	36 (14.1)	20 (15.2)	6 (15)	515 (2.9)
Autoimmune Diseases	(Yes; %)	5607 (15.6)	5383 (15.5)	224 (17.6)	60 (23.4)	34 (25.8)	7 (17.5)	1515 (8.4)
ComorbidityScore	mean (SD)	2.3 (1.5)	2.3 (1.5)	2.3 (1.5)	3.1 (1.6)	3.3 (1.7)	3.9 (1.6)	1.4 (1.2)

Abbreviations: Black, non-Hispanic Black/African American; BMI, body mass index; MM, Michigan Medicine; SES, social economics status; HS, high school; FPL, federal poverty level; White, non-Hispanic White.

Table S2. Cont'd

Non-Hispanic Blacks

Variable	Statistics	Tested	Tested Negative	Tested Positive	Hospitalized	ICU	Deceased	Unmatched Controls
n		5763	5117	646	265	139	42	2165
Age	mean (SD)	42.8 (21.7)	42.1 (22)	48.6 (18.6)	56.8 (17.1)	57.8 (17)	67.2 (11.4)	36.8 (23.2)
	median (IQR)	44 (34)	43 (35)	49 (27)	58 (24)	59 (22.5)	66.5 (17.5)	34 (38)
AgeCategory	n (%)							
[0,18)		697 (12.1)	675 (13.2)	22 (3.4)	5 (1.9)	4 (2.9)	0 (0)	547 (25.3)
[18,35)		1487 (25.8)	1355 (26.5)	132 (20.4)	21 (7.9)	11 (7.9)	0 (0)	541 (25)
[35,50)		1173 (20.4)	1001 (19.6)	172 (26.6)	63 (23.8)	23 (16.5)	3 (7.1)	376 (17.4)
[50,65)		1340 (23.3)	1157 (22.6)	183 (28.3)	83 (31.3)	50 (36)	15 (35.7)	384 (17.7)
[65,80)		890 (15.4)	784 (15.3)	106 (16.4)	67 (25.3)	39 (28.1)	15 (35.7)	249 (11.5)
[80,100)		176 (3.1)	145 (2.8)	31 (4.8)	26 (9.8)	12 (8.6)	9 (21.4)	68 (3.1)
Gender	(Male; %)	2336 (40.5)	2067 (40.4)	269 (41.6)	140 (52.8)	81 (58.3)	25 (59.5)	1011 (46.7)
Primary Care in MM	(Yes; %)	3284 (57)	2973 (58.1)	311 (48.1)	83 (31.3)	34 (24.5)	9 (21.4)	431 (19.9)
BMI	mean (SD)	31.5 (8.8)	31.1 (8.6)	34 (9.9)	34.6 (12.1)	35.4 (14.3)	33.1 (6.5)	30.7 (8.3)
BMIcategory	n (%)							
[0,18.5)		100 (2.1)	92 (2.2)	8 (1.4)	5 (2)	0 (0)	0 (0)	22 (1.7)
[18.5,25)		996 (20.6)	940 (22.1)	56 (9.6)	20 (7.8)	13 (9.8)	3 (7.1)	298 (23.7)
[25,30)		1327 (27.4)	1183 (27.8)	144 (24.8)	70 (27.5)	36 (27.1)	13 (31)	357 (28.3)
[30,200)		2421 (50)	2048 (48)	373 (64.2)	160 (62.7)	84 (63.2)	26 (61.9)	583 (46.3)
Smoker	(Ever; %)	2011 (37.4)	1831 (38)	180 (32.2)	83 (36.7)	43 (41)	19 (70.4)	517 (28.3)
SmokingStatus	n (%)							
Never		3367 (62.6)	2988 (62)	379 (67.8)	143 (63.3)	62 (59)	8 (29.6)	1312 (71.7)
Past		1350 (25.1)	1200 (24.9)	150 (26.8)	77 (34.1)	41 (39)	19 (70.4)	304 (16.6)
Current		661 (12.3)	631 (13.1)	30 (5.4)	6 (2.7)	2 (1.9)	0 (0)	213 (11.6)
Drinker	(Yes; %)	2393 (58.4)	2134 (58)	259 (62.3)	81 (57)	37 (57.8)	12 (60)	575 (44.7)
NDI	mean (SD)	0.18 (0.1)	0.18 (0.1)	0.19 (0.11)	0.2 (0.11)	0.21 (0.11)	0.21 (0.1)	0.19 (0.11)
PersonsPerSquareMile	mean (SD)	3594.3 (2406.4)	3547.9 (2407.2)	3937.7 (2374.8)	4439.2 (2482)	4555.3 (2472.3)	5050.3 (2260.1)	3541.3 (2604.8)
Respiratory Diseases	(Yes; %)	3862 (75.6)	3468 (75.4)	394 (77.7)	138 (78.9)	66 (76.7)	26 (86.7)	1123 (53.7)
Circulatory Diseases	(Yes; %)	3843 (75.2)	3446 (74.9)	397 (78.3)	151 (86.3)	77 (89.5)	29 (96.7)	969 (46.4)
Any Cancer	(Yes; %)	1210 (23.7)	1094 (23.8)	116 (22.9)	51 (29.1)	24 (27.9)	14 (46.7)	262 (12.5)
Type 2 Diabetes	(Yes; %)	1305 (25.6)	1143 (24.8)	162 (32)	80 (45.7)	48 (55.8)	23 (76.7)	267 (12.8)
Kidney Diseases	(Yes; %)	1198 (23.5)	1067 (23.2)	131 (25.8)	81 (46.3)	51 (59.3)	23 (76.7)	165 (7.9)
Liver Diseases	(Yes; %)	433 (8.5)	391 (8.5)	42 (8.3)	13 (7.4)	6 (7)	4 (13.3)	36 (1.7)
Autoimmune Diseases	(Yes; %)	1086 (21.3)	973 (21.2)	113 (22.3)	42 (24)	24 (27.9)	9 (30)	183 (8.8)
ComorbidityScore	mean (SD)	2.5 (1.6)	2.5 (1.6)	2.7 (1.6)	3.2 (1.6)	3.4 (1.6)	4.3 (1.4)	1.4 (1.3)

Abbreviations: Black, non-Hispanic Black/African American; BMI, body mass index; MM, Michigan Medicine; SES, social economics status; HS, high school; FPL, federal poverty level; White, non-Hispanic White.

Table S3. "COVID-19 Positive vs. Controls" PheWAS Merged Top 50 Traits Using Adjustment 2

TRAIT (PHECODE)	CATEGORY	RANK ALL/NHW/NHB	ALL OR (95% CI) P-VALUE	NHW OR (95% CI) P-VALUE	NHB OR (95% CI) P-VALUE
Viral infection (079)	infectious diseases	27/41/79	3.91 (3.36, 4.55) 1.36e-69	3.88 (3.22, 4.68) 9.2e-46	3.35 (2.61, 4.31) 3.72e-21
Diabetes mellitus (250)	endocrine/metabolic	35/59/35	3.34 (2.91, 3.84) 3.32e-64	3.19 (2.69, 3.79) 2.22e-40	3.39 (2.73, 4.2) 9e-29
Type 2 diabetes with renal manifestations (250.22)	endocrine/metabolic	108/129/26	6.96 (5.24, 9.24) 5.65e-41	7.96 (5.58, 11.4) 2.71e-30	8.46 (5.89, 12.1) 4.55e-31
Abnormal glucose (250.4)	endocrine/metabolic	17/12/7	4.73 (4.03, 5.54) 1.42e-81	5.04 (4.14, 6.13) 1.59e-58	5.5 (4.27, 7.09) 9.45e-40
Other abnormal glucose (250.42)	endocrine/metabolic	19/18/8	4.9 (4.15, 5.78) 2.96e-78	5.11 (4.16, 6.28) 5.17e-54	5.71 (4.39, 7.41) 4.69e-39
Vitamin deficiency (261)	endocrine/metabolic	24/17/65	4.15 (3.55, 4.85) 1.34e-71	4.69 (3.85, 5.7) 5.05e-54	3.57 (2.77, 4.59) 5.63e-23
Vitamin D deficiency (261.4)	endocrine/metabolic	25/26/55	4.23 (3.61, 4.96) 1.28e-70	4.65 (3.8, 5.69) 9.15e-51	3.68 (2.85, 4.74) 1.46e-23
Disorders of fluid, electrolyte, and acid-base balance (276)	endocrine/metabolic	18/20/15	4.17 (3.59, 4.84) 1.04e-78	4.4 (3.64, 5.31) 8.24e-53	4.54 (3.58, 5.75) 9.8e-36
Electrolyte imbalance (276.1)	endocrine/metabolic	20/30/11	5.25 (4.41, 6.25) 2.76e-77	5.21 (4.18, 6.48) 3.21e-49	5.78 (4.4, 7.58) 9.91e-37
Hyperpotassemia (276.13)	endocrine/metabolic	67/66/48	6.54 (5.11, 8.37) 2.33e-50	6.98 (5.21, 9.34) 7.15e-39	7.41 (5.09, 10.8) 1.62e-25
Hypovolemia (276.5)	endocrine/metabolic	63/43/53	4.46 (3.66, 5.42) 7.74e-51	5.35 (4.22, 6.78) 1.62e-43	4.94 (3.64, 6.71) 1.07e-24
Overweight, obesity and other hyperalimentation (278)	endocrine/metabolic	6/13/17	4.15 (3.65, 4.71) 3.18e-106	3.81 (3.23, 4.49) 3.8e-57	3.89 (3.14, 4.83) 2.77e-35
Obesity (278.1)	endocrine/metabolic	7/31/12	4.19 (3.66, 4.79) 1.21e-95	3.75 (3.14, 4.47) 4.71e-49	4.11 (3.3, 5.12) 1.89e-36
Morbid obesity (278.11)	endocrine/metabolic	12/40/4	5.56 (4.69, 6.59) 4.09e-87	4.91 (3.94, 6.11) 7.86e-46	5.54 (4.3, 7.13) 3.23e-40
Iron deficiency anemias (280)	hematopoietic	34/65/22	4.94 (4.1, 5.94) 2.38e-64	4.9 (3.86, 6.23) 7.1e-39	5.11 (3.91, 6.68) 7.35e-33
Iron deficiency anemias, unspecified or not due to blood loss (280.1)	hematopoietic	37/81/20	4.93 (4.08, 5.94) 1.52e-62	4.81 (3.76, 6.14) 3.1e-36	5.2 (3.97, 6.8) 3.56e-33
Other anemias (285)	hematopoietic	40/48/58	3.2 (2.78, 3.69) 4.8e-58	3.54 (2.95, 4.24) 2.38e-42	3.13 (2.5, 3.91) 1.89e-23
Anemia of chronic disease (285.2)	hematopoietic	114/49/134	6.66 (5.03, 8.82) 5.44e-40	9.2 (6.67, 12.7) 9.05e-42	5.75 (3.83, 8.65) 4.29e-17
Diseases of white blood cells (288)	hematopoietic	69/99/34	4.5 (3.69, 5.48) 3.97e-50	4.58 (3.57, 5.87) 2.46e-33	5.73 (4.22, 7.78) 4.75e-29
Elevated white blood cell count (288.2)	hematopoietic	88/125/38	4.73 (3.82, 5.85) 2.2e-46	4.85 (3.71, 6.35) 1.11e-30	6.2 (4.47, 8.6) 6.44e-28
Other specified nonpsychotic and/or transient mental disorders (291)	mental disorders	83/34/256	4.9 (3.95, 6.08) 3.45e-47	6.39 (4.98, 8.2) 2.91e-48	3.55 (2.47, 5.11) 8.79e-12
Alteration of consciousness (291.8)	mental disorders	136/45/321	5.05 (3.94, 6.48) 4.2e-37	7.26 (5.48, 9.63) 4.14e-43	3.68 (2.43, 5.59) 9.01e-10
Adjustment reaction (304)	mental disorders	59/44/406	5.09 (4.11, 6.29) 4.23e-51	5.89 (4.58, 7.57) 1.76e-43	2.68 (1.87, 3.86) 9.56e-08
Sleep disorders (327)	neurological	39/60/32	3.07 (2.68, 3.51) 5.36e-60	3.15 (2.66, 3.73) 3.82e-40	3.59 (2.87, 4.49) 2.84e-29
Sleep apnea (327.3)	neurological	117/233/40	2.91 (2.48, 3.41) 1.14e-39	2.77 (2.25, 3.4) 2.85e-22	3.96 (3.09, 5.07) 1.14e-27
Obstructive sleep apnea (327.32)	neurological	80/144/23	3.59 (3.02, 4.26) 3.45e-48	3.48 (2.8, 4.34) 5.57e-29	5.05 (3.86, 6.6) 1.99e-32
Insomnia (327.4)	neurological	89/42/168	3.94 (3.26, 4.75) 2.63e-46	4.74 (3.81, 5.89) 2.51e-44	3.72 (2.68, 5.17) 4.32e-15

Abbreviations: NHB: non-Hispanic Black/African American; NHW: non-Hispanic Whites; OR: odds ratio; CI: confidence interval; n/a: not available

Table S3 cont'd

TRAIT (PHECODE)	CATEGORY	RANK ALL/NHW/NHB	ALL OR (95% CI) P-VALUE	NHW OR (95% CI) P-VALUE	NHB OR (95% CI) P-VALUE
Pain (338)	neurological	56/154/44	2.78 (2.44, 3.17) 2.69e-52	2.59 (2.19, 3.07) 3.66e-28	3.34 (2.68, 4.15) 2.51e-27
Chronic pain (338.2)	neurological	47/102/30	3.31 (2.85, 3.84) 3.17e-55	3.22 (2.66, 3.9) 6.19e-33	3.99 (3.15, 5.06) 3.32e-30
Dizziness and giddiness (Light-headedness and vertigo) (386.9)	sense organs	45/38/52	3.6 (3.07, 4.22) 2.19e-56	4.1 (3.38, 4.98) 3.64e-46	3.89 (3.01, 5.05) 8.19e-25
Hypertensive heart and/or renal disease (401.2)	circulatory system	142/77/25	4.97 (3.87, 6.37) 1.39e-36	6.44 (4.83, 8.59) 9.61e-37	6.94 (5.01, 9.61) 2.16e-31
Nonspecific chest pain (418)	circulatory system	8/6/9	4 (3.5, 4.56) 7.9e-95	4.36 (3.69, 5.16) 3.13e-67	4.13 (3.32, 5.13) 2.25e-37
Cardiac conduction disorders (426)	circulatory system	50/37/61	3.56 (3.04, 4.18) 1.53e-54	4.13 (3.41, 5.01) 5.47e-47	3.53 (2.75, 4.53) 3.53e-23
Abnormal electrocardiogram [ECG] [EKG] (426.7)	circulatory system	43/32/63	3.82 (3.24, 4.51) 8.91e-57	4.49 (3.67, 5.49) 7.25e-49	3.65 (2.82, 4.72) 5.11e-23
Cardiac dysrhythmias (427)	circulatory system	23/36/19	3.46 (3.02, 3.96) 7.36e-73	3.52 (2.97, 4.18) 6.97e-48	3.94 (3.15, 4.93) 1.89e-33
Other specified cardiac dysrhythmias (427.3)	circulatory system	82/103/41	3.71 (3.11, 4.44) 3.4e-47	3.97 (3.17, 4.98) 7.17e-33	4.63 (3.51, 6.1) 1.2e-27
Tachycardia NOS (427.7)	circulatory system	22/35/33	6.01 (4.96, 7.29) 4.02e-74	5.96 (4.69, 7.57) 3.15e-48	5.57 (4.12, 7.52) 4.26e-29
Ill-defined descriptions and complications of heart disease (429)	circulatory system	9/7/21	4.26 (3.71, 4.89) 8.12e-94	4.51 (3.8, 5.36) 4.92e-66	3.99 (3.18, 5.01) 4.48e-33
Abnormal function study of cardiovascular system (429.2)	circulatory system	31/21/31	5.04 (4.2, 6.05) 6.76e-68	5.62 (4.5, 7.02) 1.71e-52	4.86 (3.69, 6.39) 1.25e-29
Symptoms involving cardiovascular system (429.3)	circulatory system	16/10/27	4.43 (3.8, 5.15) 6e-82	4.69 (3.89, 5.65) 3.15e-59	4.37 (3.4, 5.61) 1.21e-30
Acute sinusitis (464)	respiratory	36/22/86	5.08 (4.21, 6.14) 1.69e-63	5.51 (4.42, 6.88) 7.81e-52	5.06 (3.59, 7.12) 1.73e-20
Acute upper respiratory infections of multiple or unspecified sites (465)	respiratory	10/11/72	4.04 (3.52, 4.62) 7.44e-90	4 (3.38, 4.73) 4.65e-59	3.04 (2.43, 3.81) 2.57e-22
Pneumonia (480)	respiratory	4/4/2	5.82 (4.98, 6.8) 2.47e-108	6.08 (4.99, 7.4) 2.77e-72	6.8 (5.23, 8.83) 8.86e-47
Viral pneumonia (480.2)	respiratory	21/14/13	85.7 (53.5, 137) 1.87e-76	51.1 (31.4, 82.9) 5.43e-57	60.5 (31.9, 115) 2.94e-36
Respiratory failure, insufficiency, arrest (509)	respiratory	44/98/14	4.86 (3.99, 5.9) 1.3e-56	4.95 (3.81, 6.42) 2.25e-33	6.67 (4.96, 8.97) 3.2e-36
Respiratory failure (509.1)	respiratory	38/64/29	7.49 (5.89, 9.52) 6.52e-61	7.75 (5.7, 10.5) 2.99e-39	7.97 (5.59, 11.4) 2.21e-30
Other symptoms of respiratory system (512)	respiratory	3/3/3	4.4 (3.88, 4.99) 3.95e-119	4.75 (4.04, 5.59) 5.48e-78	4.56 (3.66, 5.68) 4.23e-42
Painful respiration (512.2)	respiratory	30/23/24	8.82 (6.91, 11.3) 5.39e-68	9.91 (7.36, 13.3) 1.17e-51	9.03 (6.26, 13) 4.83e-32
Shortness of breath (512.7)	respiratory	1/2/1	6.25 (5.38, 7.25) 1.89e-127	6.72 (5.55, 8.15) 6.83e-84	6.28 (4.91, 8.02) 6.6e-49
Cough (512.8)	respiratory	2/1/5	5.37 (4.66, 6.19) 2.39e-119	6 (5.01, 7.19) 3.73e-84	5.35 (4.17, 6.86) 6.44e-40
Other dyspnea (512.9)	respiratory	11/9/10	4.74 (4.06, 5.52) 1.95e-88	5.43 (4.46, 6.6) 4.86e-64	5.32 (4.11, 6.89) 8.9e-37
Respiratory abnormalities (513)	respiratory	5/5/6	4.38 (3.84, 4.99) 5.64e-108	4.47 (3.78, 5.29) 2.88e-68	4.39 (3.53, 5.47) 7.42e-40
Diseases of esophagus (530)	digestive	96/148/36	2.61 (2.28, 2.99) 7.46e-44	2.63 (2.21, 3.12) 1.37e-28	3.55 (2.84, 4.45) 2.07e-28
Esophagitis, GERD and related diseases (530.1)	digestive	97/155/39	2.62 (2.29, 3.01) 1.89e-43	2.63 (2.22, 3.13) 3.7e-28	3.53 (2.82, 4.43) 9.69e-28

Abbreviations: NHB: non-Hispanic Black/African American; NHW: non-Hispanic Whites; OR: odds ratio; CI: confidence interval; n/a: not available

Table S3 cont'd

TRAIT (PHECODE)	CATEGORY	RANK ALL/NHW/NHB	ALL OR (95% CI) P-VALUE	NHW OR (95% CI) P-VALUE	NHB OR (95% CI) P-VALUE
GERD (530.11)	digestive	95/156/42	2.66 (2.31, 3.05) 7.28e-44	2.66 (2.23, 3.16) 3.96e-28	3.54 (2.82, 4.45) 1.26e-27
Abnormal serum enzyme levels (573.9)	digestive	76/128/28	4.24 (3.5, 5.14) 2.76e-49	4.26 (3.32, 5.46) 2.28e-30	5.56 (4.15, 7.45) 1.31e-30
Renal failure (585)	genitourinary	64/62/37	3.75 (3.15, 4.46) 8.46e-51	4.19 (3.39, 5.19) 1.25e-39	4.05 (3.16, 5.2) 2.8e-28
Acute renal failure (585.1)	genitourinary	33/19/16	6.2 (5.04, 7.63) 7.5e-67	7.28 (5.65, 9.37) 1.38e-53	6.6 (4.9, 8.88) 1.21e-35
Chronic Kidney Disease, Stage III (585.33)	genitourinary	166/138/49	4.8 (3.73, 6.18) 3.72e-34	5.6 (4.15, 7.55) 1.66e-29	6.64 (4.65, 9.49) 2.26e-25
Hematuria (593)	genitourinary	81/50/123	4.3 (3.54, 5.24) 4.81e-48	5.12 (4.04, 6.48) 9.51e-42	4.33 (3.11, 6.04) 4.61e-18
Other symptoms/disorders or the urinary system (599)	genitourinary	29/33/54	3.33 (2.91, 3.82) 4.09e-68	3.57 (3.01, 4.23) 1.75e-48	3.26 (2.59, 4.1) 4.84e-24
Noninflammatory female genital disorders (619)	genitourinary	42/119/155	4.35 (3.63, 5.21) 2.5e-57	4.05 (3.2, 5.11) 1.51e-31	3.22 (2.42, 4.27) 7.45e-16
Disorders of menstruation and other abnormal bleeding from female genital tract (626)	genitourinary	48/120/197	4.3 (3.58, 5.16) 3.2e-55	4.11 (3.24, 5.22) 1.67e-31	3.08 (2.3, 4.13) 5.12e-14
Superficial cellulitis and abscess (681)	dermatologic	49/25/195	3.97 (3.34, 4.73) 4.12e-55	4.76 (3.88, 5.83) 7.23e-51	3.13 (2.33, 4.22) 4.43e-14
Peripheral enthesopathies and allied syndromes (726)	musculoskeletal	32/27/47	4.09 (3.49, 4.8) 3.87e-67	4.44 (3.65, 5.4) 1.63e-50	4.22 (3.23, 5.53) 1.08e-25
Other disorders of soft tissues (729)	musculoskeletal	55/29/81	5.71 (4.56, 7.14) 2.27e-52	7.52 (5.76, 9.83) 1.6e-49	5.48 (3.84, 7.81) 5.13e-21
Symptoms and disorders of the joints (741)	musculoskeletal	68/61/43	3.39 (2.89, 3.98) 2.72e-50	3.85 (3.15, 4.7) 1.1e-39	4.2 (3.24, 5.44) 1.42e-27
Pain in joint (745)	musculoskeletal	28/15/100	3.05 (2.69, 3.46) 6.29e-69	3.71 (3.15, 4.36) 1.41e-56	2.69 (2.17, 3.33) 1.54e-19
Back pain (760)	symptoms	26/28/119	3.21 (2.83, 3.66) 1.82e-70	3.42 (2.91, 4.03) 1.39e-49	2.63 (2.12, 3.27) 2.79e-18
Swelling of limb (771.1)	symptoms	46/39/46	4.52 (3.74, 5.45) 1.34e-55	5.31 (4.22, 6.68) 4.99e-46	4.55 (3.43, 6.04) 1.04e-25
Edema (782.3)	symptoms	110/47/161	3.75 (3.09, 4.55) 9.52e-41	4.75 (3.8, 5.94) 1.74e-42	3.47 (2.56, 4.7) 1.24e-15

Abbreviations: NHB: non-Hispanic Black/African American; NHW: non-Hispanic Whites; OR: odds ratio; CI: confidence interval; n/a: not available

Table S4 "Hospitalized vs. Alive & Not hospitalized" PheWAS Merged Top 50 Traits Using Adjustment 2

TRAIT (PHECODE)	CATEGORY	RANK ALL/NHW/NHB	ALL OR (95% CI) P-VALUE	NHW OR (95% CI) P-VALUE	NHB OR (95% CI) P-VALUE
Septicemia (038)	infectious diseases	12/62/8	4.32 (2.65, 7.04) 4.08e-09	2.95 (1.55, 5.61) 0.000948	7.25 (2.6, 20.2) 0.000151
Bacteremia (038.3)	infectious diseases	40/117/18	5.98 (2.69, 13.3) 1.17e-05	4.31 (1.56, 11.9) 0.00471	11.9 (2.48, 57.4) 0.002
Streptococcus infection (041.2)	infectious diseases	46/43/51	4.69 (2.24, 9.84) 4.26e-05	6.66 (2.36, 18.8) 0.000346	4.11 (1.24, 13.7) 0.0211
Protein-calorie malnutrition (260)	endocrine/metabolic	79/31/518	2.54 (1.49, 4.33) 0.000584	4.02 (1.96, 8.21) 0.000138	1.21 (0.494, 2.98) 0.672
Disorders of carbohydrate transport and metabolism (271)	endocrine/metabolic	155/248/36	0.537 (0.349, 0.827) 0.00473	0.497 (0.253, 0.976) 0.0424	0.412 (0.21, 0.809) 0.01
Intestinal disaccharidase deficiencies and disaccharide malabsorption (271.3)	endocrine/metabolic	156/249/37	0.537 (0.349, 0.827) 0.00473	0.497 (0.253, 0.976) 0.0424	0.412 (0.21, 0.809) 0.01
Disorders of phosphorus metabolism (275.53)	endocrine/metabolic	35/30/70	4.3 (2.28, 8.12) 6.62e-06	6.46 (2.49, 16.7) 0.000122	2.87 (1.08, 7.57) 0.0337
Disorders of fluid, electrolyte, and acid-base balance (276)	endocrine/metabolic	21/3/173	2.18 (1.62, 2.93) 2.52e-07	3.47 (2.28, 5.29) 6.16e-09	1.47 (0.894, 2.42) 0.128
Electrolyte imbalance (276.1)	endocrine/metabolic	24/4/125	2.34 (1.68, 3.26) 5.55e-07	4.02 (2.48, 6.51) 1.5e-08	1.68 (0.959, 2.94) 0.0699
Hyperosmolality and/or hypernatremia (276.11)	endocrine/metabolic	41/160/103	20.5 (5.07, 83.2) 2.31e-05	66.8 (2.6, 1720) 0.0112	18.4 (0.925, 366) 0.0563
Hyposmolality and/or hyponatremia (276.12)	endocrine/metabolic	34/9/203	3.29 (1.97, 5.51) 5.86e-06	6.38 (3.09, 13.2) 5.66e-07	1.82 (0.78, 4.25) 0.166
Hyperpotassemia (276.13)	endocrine/metabolic	38/19/56	2.78 (1.76, 4.38) 1.02e-05	3.92 (2.09, 7.34) 1.99e-05	2.45 (1.12, 5.32) 0.0241
Acid-base balance disorder (276.4)	endocrine/metabolic	7/6/13	7.19 (3.91, 13.3) 2.42e-10	9.23 (3.94, 21.6) 3.03e-07	5.64 (2.08, 15.3) 0.000686
Acidosis (276.41)	endocrine/metabolic	11/11/22	6.65 (3.54, 12.5) 3.78e-09	7.77 (3.29, 18.4) 2.95e-06	4.91 (1.77, 13.6) 0.0022
Hypovolemia (276.5)	endocrine/metabolic	55/12/391	2.16 (1.47, 3.19) 9.75e-05	3.4 (2.02, 5.71) 3.89e-06	1.26 (0.677, 2.36) 0.463
Fluid overload (276.6)	endocrine/metabolic	33/96/24	5.83 (2.75, 12.4) 4.29e-06	5.04 (1.73, 14.7) 0.00304	5.11 (1.74, 14.9) 0.00292
Immunity deficiency (279.1)	endocrine/metabolic	134/48/546	2.84 (1.42, 5.7) 0.00323	6.41 (2.26, 18.2) 0.00047	1.2 (0.432, 3.33) 0.727
Other anemias (285)	hematopoietic	113/44/280	1.59 (1.19, 2.13) 0.00194	2.1 (1.4, 3.15) 0.000363	1.31 (0.809, 2.11) 0.274
Acute posthemorrhagic anemia (285.1)	hematopoietic	39/122/44	3.22 (1.91, 5.42) 1.08e-05	2.83 (1.36, 5.89) 0.00548	2.84 (1.26, 6.43) 0.0119
Anemia of chronic disease (285.2)	hematopoietic	22/21/30	3.98 (2.34, 6.75) 3.16e-07	4.15 (2.11, 8.15) 3.71e-05	3.81 (1.51, 9.66) 0.00472
Anemia in chronic kidney disease (285.21)	hematopoietic	124/303/35	2.82 (1.43, 5.56) 0.0027	2.27 (0.9, 5.72) 0.0825	5.04 (1.48, 17.2) 0.00975
Diseases of white blood cells (288)	hematopoietic	49/57/52	2.2 (1.5, 3.22) 5.81e-05	2.47 (1.46, 4.19) 0.000782	2.05 (1.11, 3.79) 0.0215
Aphasia/speech disturbance (292.1)	mental disorders	98/46/406	2.9 (1.52, 5.55) 0.00127	3.95 (1.83, 8.52) 0.000458	1.61 (0.408, 6.31) 0.498
Epilepsy, recurrent seizures, convulsions (345)	neurological	131/25/424	2.22 (1.31, 3.75) 0.00296	4.17 (2.07, 8.41) 6.39e-05	1.35 (0.514, 3.57) 0.539
Epilepsy (345.1)	neurological	223/27/NA	2.75 (1.21, 6.26) 0.0159	11.2 (3.29, 38.3) 0.000114	
Convulsions (345.3)	neurological	169/33/381	2.16 (1.25, 3.74) 0.0058	4 (1.92, 8.35) 0.000223	1.47 (0.543, 3.96) 0.451

Abbreviations: NHB: non-Hispanic Black/African American; NHW: non-Hispanic Whites; OR: odds ratio; CI: confidence interval; n/a: not available

Table S4 cont'd

TRAIT (PHECODE)	CATEGORY	RANK ALL/NHW/NHB	ALL OR (95% CI) P-VALUE	NHW OR (95% CI) P-VALUE	NHB OR (95% CI) P-VALUE
Hypermetropia (367.8)	sense organs	96/352/48	0.398 (0.228, 0.697) 0.00126	0.554 (0.261, 1.18) 0.124	0.342 (0.139, 0.842) 0.0196
Hypertension (401)	circulatory system	26/51/87	2.07 (1.54, 2.78) 1.52e-06	2.08 (1.37, 3.14) 0.000522	1.69 (1.01, 2.85) 0.0473
Essential hypertension (401.1)	circulatory system	30/61/85	2.05 (1.52, 2.76) 2.53e-06	2.02 (1.33, 3.06) 0.000945	1.71 (1.01, 2.89) 0.0458
Hypertensive heart and/or renal disease (401.2)	circulatory system	9/14/12	4.6 (2.82, 7.52) 1.03e-09	4.62 (2.35, 9.11) 9.64e-06	4.06 (1.83, 9) 0.000553
Hypertensive chronic kidney disease (401.22)	circulatory system	25/40/16	4.53 (2.49, 8.23) 7.13e-07	4.78 (2.05, 11.1) 0.000287	4.79 (1.87, 12.3) 0.00109
Other hypertensive complications (401.3)	circulatory system	32/47/39	3.34 (2.01, 5.56) 3.57e-06	3.89 (1.82, 8.31) 0.000461	2.75 (1.27, 5.97) 0.0105
Pulmonary heart disease (415)	circulatory system	28/22/49	3.36 (2.05, 5.52) 1.62e-06	4.45 (2.18, 9.1) 4.27e-05	2.27 (1.14, 4.55) 0.0202
Chronic pulmonary heart disease (415.2)	circulatory system	36/49/31	4.36 (2.29, 8.3) 7.22e-06	6.51 (2.28, 18.6) 0.000475	3.14 (1.39, 7.08) 0.00575
Abnormal electrocardiogram [ECG] [EKG] (426.7)	circulatory system	86/36/320	1.74 (1.26, 2.42) 0.000829	2.37 (1.49, 3.76) 0.000258	1.31 (0.757, 2.26) 0.336
Tachycardia NOS (427.7)	circulatory system	29/10/94	2.55 (1.74, 3.74) 1.63e-06	3.5 (2.08, 5.89) 2.39e-06	1.87 (0.996, 3.53) 0.0515
Congestive heart failure; nonhypertensive (428)	circulatory system	43/45/147	2.51 (1.63, 3.85) 2.79e-05	2.94 (1.62, 5.34) 0.000392	1.83 (0.904, 3.71) 0.093
Congestive heart failure (CHF) NOS (428.1)	circulatory system	50/60/166	2.55 (1.61, 4.03) 6.38e-05	2.85 (1.53, 5.32) 0.000944	1.84 (0.857, 3.96) 0.118
Abnormal function study of cardiovascular system (429.2)	circulatory system	90/41/600	1.78 (1.26, 2.5) 0.000952	2.45 (1.51, 3.98) 0.00029	1.06 (0.603, 1.88) 0.831
Peripheral vascular disease (443)	circulatory system	132/38/576	2.36 (1.34, 4.17) 0.00307	3.74 (1.83, 7.62) 0.000284	1.18 (0.391, 3.55) 0.771
Other specified peripheral vascular diseases (443.8)	circulatory system	117/37/656	2.71 (1.43, 5.14) 0.00231	4.56 (2.02, 10.3) 0.000261	0.95 (0.297, 3.04) 0.931
Peripheral vascular disease, unspecified (443.9)	circulatory system	69/26/616	3.87 (1.88, 7.96) 0.000238	6.53 (2.58, 16.5) 7.51e-05	1.11 (0.328, 3.77) 0.865
Hypotension (458)	circulatory system	15/17/14	3.4 (2.22, 5.21) 2.04e-08	3.44 (1.96, 6.04) 1.69e-05	4.12 (1.81, 9.4) 0.000753
Iatrogenic hypotension (458.2)	circulatory system	149/387/47	4.69 (1.63, 13.5) 0.00424	2.55 (0.696, 9.31) 0.158	12 (1.52, 94.8) 0.0185
Hypotension NOS (458.9)	circulatory system	19/23/27	3.3 (2.11, 5.15) 1.45e-07	3.34 (1.86, 6) 5.4e-05	3.56 (1.53, 8.3) 0.00324
Other disorders of circulatory system (459)	circulatory system	37/35/34	2.34 (1.61, 3.4) 8.69e-06	2.6 (1.56, 4.33) 0.000241	2.26 (1.26, 4.05) 0.00635
Acute upper respiratory infections of multiple or unspecified sites (465)	respiratory	94/211/43	0.627 (0.473, 0.83) 0.00111	0.645 (0.439, 0.948) 0.0255	0.537 (0.332, 0.87) 0.0116
Allergic rhinitis (476)	respiratory	67/351/41	0.547 (0.397, 0.754) 0.000228	0.717 (0.471, 1.09) 0.122	0.478 (0.271, 0.843) 0.0107
Pneumonia (480)	respiratory	20/24/65	2.28 (1.67, 3.11) 1.79e-07	2.45 (1.59, 3.79) 5.48e-05	1.79 (1.06, 3.04) 0.0308
Viral pneumonia (480.2)	respiratory	6/28/5	7.46 (4.13, 13.5) 2.56e-11	5.33 (2.28, 12.5) 0.000116	7.97 (2.75, 23.1) 0.000129
Pulmonary congestion and hypostasis (503)	respiratory	48/129/38	8.35 (2.98, 23.4) 5.33e-05	5.41 (1.61, 18.1) 0.0062	12 (1.81, 80) 0.0101
Other pulmonary inflamation or edema (505)	respiratory	27/34/25	7.08 (3.18, 15.7) 1.58e-06	8.82 (2.77, 28.1) 0.00023	5.87 (1.83, 18.8) 0.00293
Empyema and pneumothorax (506)	respiratory	23/93/10	4.55 (2.54, 8.14) 3.3e-07	3.36 (1.52, 7.41) 0.00273	6.81 (2.46, 18.9) 0.000226

Abbreviations: NHB: non-Hispanic Black/African American; NHW: non-Hispanic Whites; OR: odds ratio; CI: confidence interval; n/a: not available

Table S4 cont'd

TRAIT (PHECODE)	CATEGORY	RANK ALL/NHW/NHB	ALL OR (95% CI) P-VALUE	NHW OR (95% CI) P-VALUE	NHB OR (95% CI) P-VALUE
Pleurisy; pleural effusion (507)	respiratory	16/15/33	4.43 (2.62, 7.5) 2.99e-08	5.66 (2.61, 12.3) 1.11e-05	3.07 (1.38, 6.81) 0.00579
Pulmonary collapse; interstitial and compensatory emphysema (508)	respiratory	18/7/218	5.97 (3.14, 11.3) 4.72e-08	11.8 (4.57, 30.4) 3.36e-07	1.94 (0.733, 5.11) 0.182
Respiratory failure, insufficiency, arrest (509)	respiratory	1/2/1	6.78 (4.51, 10.2) 4.3e-20	5.45 (3.09, 9.64) 5.26e-09	7.1 (3.56, 14.1) 2.62e-08
Respiratory failure (509.1)	respiratory	2/8/2	8.64 (5.24, 14.2) 2.62e-17	5.45 (2.83, 10.5) 3.98e-07	10.8 (4.29, 27.2) 4.38e-07
Respiratory insufficiency (509.2)	respiratory	5/13/17	7.05 (3.98, 12.5) 2.14e-11	7 (3.03, 16.1) 5.08e-06	4.26 (1.72, 10.5) 0.00171
Pulmonary insufficiency or respiratory failure following trauma and surgery (509.3)	respiratory	10/50/3	14.5 (5.99, 34.9) 2.79e-09	12.4 (3.02, 50.6) 0.000477	19.1 (4.42, 82.6) 7.8e-05
Dependence on respirator [Ventilator] or supplemental oxygen (509.8)	respiratory	8/18/6	8.15 (4.2, 15.8) 6.01e-10	7.95 (3.09, 20.5) 1.74e-05	8.23 (2.79, 24.2) 0.000133
Other diseases of lung (510)	respiratory	270/42/327	1.55 (1.03, 2.32) 0.0336	2.76 (1.58, 4.82) 0.000343	0.728 (0.375, 1.41) 0.348
Cough (512.8)	respiratory	766/452/40	0.89 (0.657, 1.21) 0.451	1.29 (0.854, 1.94) 0.228	0.489 (0.282, 0.846) 0.0106
Dysphagia (532)	digestive	187/39/657	1.7 (1.14, 2.53) 0.00864	2.68 (1.57, 4.57) 0.000285	0.972 (0.49, 1.93) 0.936
Renal failure (585)	genitourinary	4/5/4	3.32 (2.36, 4.66) 4.55e-12	3.67 (2.28, 5.92) 8.95e-08	3.11 (1.76, 5.48) 9e-05
Acute renal failure (585.1)	genitourinary	3/1/9	4.33 (2.9, 6.45) 6.06e-13	5.42 (3.08, 9.57) 5.22e-09	3.44 (1.79, 6.59) 0.000202
Chronic renal failure [CKD] (585.3)	genitourinary	13/20/7	3.08 (2.11, 4.49) 5.82e-09	3.13 (1.85, 5.28) 1.99e-05	3.42 (1.81, 6.46) 0.000147
End stage renal disease (585.32)	genitourinary	57/73/50	3.67 (1.9, 7.06) 0.000102	6.83 (2.15, 21.8) 0.00114	2.83 (1.18, 6.83) 0.0204
Chronic Kidney Disease, Stage III (585.33)	genitourinary	31/29/23	3.26 (1.99, 5.34) 2.81e-06	3.73 (1.91, 7.3) 0.000121	3.6 (1.58, 8.19) 0.00222
Chronic Kidney Disease, Stage IV (585.34)	genitourinary	53/126/28	3.99 (2.01, 7.93) 8.01e-05	3.92 (1.48, 10.4) 0.00602	5.34 (1.73, 16.4) 0.00351
Chronic kidney disease, Stage I or II (585.4)	genitourinary	42/226/11	3.98 (2.09, 7.55) 2.45e-05	2.82 (1.09, 7.32) 0.0329	6.33 (2.3, 17.4) 0.000358
Other disorders of the kidney and ureters (586)	genitourinary	45/16/90	2.12 (1.48, 3.04) 4.08e-05	2.97 (1.81, 4.86) 1.53e-05	1.83 (1, 3.33) 0.0488

Abbreviations: NHB: non-Hispanic Black/African American; NHW: non-Hispanic Whites; OR: odds ratio; CI: confidence interval; n/a: not available

Table S5 "ICU & Dead vs. Alive & no ICU Stay" Merged PheWAS Top 50 Traits Using Adjustment 2

TRAIT (PHECODE)	CATEGORY	RANK ALL/NHW/NHB	ALL OR (95% CI) P-VALUE	NHW OR (95% CI) P-VALUE	NHB OR (95% CI) P-VALUE
Septicemia (038)	infectious diseases	8/38/12	4.73 (3, 7.47) 2.31e-11	3.74 (1.94, 7.2) 7.83e-05	4.58 (2.12, 9.9) 0.000107
Bacteremia (038.3)	infectious diseases	17/31/13	8.91 (4.27, 18.6) 5.79e-09	7.85 (2.89, 21.3) 5.21e-05	10.3 (3.07, 34.9) 0.000163
Staphylococcus infections (041.1)	infectious diseases	103/20/662	3.52 (1.76, 7.04) 0.000368	7.38 (3, 18.2) 1.41e-05	0.93 (0.199, 4.34) 0.926
Methicillin sensitive Staphylococcus aureus (041.11)	infectious diseases	101/32/NA	4.6 (1.99, 10.6) 0.000361	9.69 (3.22, 29.2) 5.37e-05	
Streptococcus infection (041.2)	infectious diseases	56/98/49	4.44 (2.19, 9.03) 3.79e-05	4.9 (1.88, 12.8) 0.00117	5.64 (1.69, 18.9) 0.005
Protein-calorie malnutrition (260)	endocrine/metabolic	62/11/270	2.94 (1.75, 4.96) 5.13e-05	5.37 (2.7, 10.7) 1.59e-06	1.72 (0.69, 4.3) 0.243
Proteinuria (269)	endocrine/metabolic	337/606/50	1.71 (1.01, 2.88) 0.0459	1.36 (0.613, 3) 0.452	2.96 (1.38, 6.37) 0.00538
Disorders of mineral metabolism (275)	endocrine/metabolic	28/77/30	2.85 (1.9, 4.27) 4.34e-07	2.67 (1.53, 4.67) 0.000541	2.9 (1.49, 5.65) 0.00171
Disorders of calcium/phosphorus metabolism (275.5)	endocrine/metabolic	39/69/106	2.77 (1.78, 4.31) 6.02e-06	2.98 (1.63, 5.43) 0.000372	2.29 (1.08, 4.84) 0.0302
Disorders of phosphorus metabolism (275.53)	endocrine/metabolic	31/26/78	4.08 (2.31, 7.22) 1.33e-06	5.85 (2.51, 13.6) 4.2e-05	2.86 (1.21, 6.77) 0.017
Disorders of fluid, electrolyte, and acid-base balance (276)	endocrine/metabolic	21/2/101	2.51 (1.8, 3.48) 4.39e-08	3.65 (2.28, 5.83) 6.26e-08	1.84 (1.06, 3.17) 0.0292
Electrolyte imbalance (276.1)	endocrine/metabolic	22/1/122	2.69 (1.88, 3.85) 6.75e-08	4.36 (2.6, 7.32) 2.61e-08	1.84 (1.01, 3.35) 0.0451
Hyperosmolality and/or hypernatremia (276.11)	endocrine/metabolic	20/157/29	11.1 (4.7, 26.4) 4.23e-08	7.05 (1.78, 27.9) 0.00538	19.5 (3.11, 123) 0.00153
Hyposmolality and/or hyponatremia (276.12)	endocrine/metabolic	82/7/486	2.7 (1.62, 4.5) 0.000147	6 (3, 12) 4.06e-07	1.26 (0.523, 3.03) 0.607
Hyperpotassemia (276.13)	endocrine/metabolic	54/62/46	2.67 (1.68, 4.23) 3.05e-05	3.43 (1.78, 6.62) 0.000232	2.95 (1.4, 6.21) 0.00443
Acid-base balance disorder (276.4)	endocrine/metabolic	4/4/4	7.53 (4.46, 12.7) 3.93e-14	7.69 (3.63, 16.3) 9.8e-08	7.06 (3.06, 16.3) 4.59e-06
Acidosis (276.41)	endocrine/metabolic	6/10/8	7.47 (4.32, 12.9) 6.14e-13	7.1 (3.26, 15.5) 8.26e-07	6.72 (2.81, 16.1) 1.9e-05
Hypovolemia (276.5)	endocrine/metabolic	77/28/182	2.28 (1.5, 3.47) 0.000123	3.24 (1.84, 5.69) 4.4e-05	1.77 (0.89, 3.53) 0.104
Fluid overload (276.6)	endocrine/metabolic	25/67/19	5.35 (2.82, 10.2) 2.78e-07	6.17 (2.27, 16.7) 0.000354	4.95 (2.02, 12.2) 0.000488
Immunity deficiency (279.1)	endocrine/metabolic	73/40/183	3.91 (1.97, 7.75) 9.19e-05	7.29 (2.69, 19.8) 9.45e-05	2.34 (0.84, 6.5) 0.104
Aplastic anemia (284)	hematopoietic	141/50/NA	4.5 (1.75, 11.6) 0.00183	7.83 (2.7, 22.8) 0.000156	
Other anemias (285)	hematopoietic	58/16/333	2.02 (1.44, 2.83) 4.09e-05	3.02 (1.89, 4.82) 3.56e-06	1.3 (0.753, 2.24) 0.348
Acute posthemorrhagic anemia (285.1)	hematopoietic	45/52/148	3.22 (1.92, 5.42) 1.02e-05	4.3 (2.01, 9.18) 0.000166	2.13 (0.95, 4.78) 0.0663
Anemia of chronic disease (285.2)	hematopoietic	30/37/48	3.6 (2.16, 5.98) 8.33e-07	4.02 (2.02, 8) 7.57e-05	3.29 (1.44, 7.49) 0.00465
Anemia in neoplastic disease (285.22)	hematopoietic	76/49/NA	9.58 (3.03, 30.3) 0.000119	15.2 (3.73, 61.5) 0.000144	
Encounter for long-term (current) use of anticoagulants (286.2)	hematopoietic	49/121/34	3.08 (1.85, 5.14) 1.65e-05	3.08 (1.49, 6.37) 0.00246	3.39 (1.54, 7.46) 0.00239

Abbreviations: NHB: non-Hispanic Black/African American; NHW: non-Hispanic Whites; OR: odds ratio; CI: confidence interval; n/a: not available

Table S5 cont'd

TRAIT (PHECODE)	CATEGORY	RANK ALL/NHW/NHB	ALL OR (95% CI) P-VALUE	NHW OR (95% CI) P-VALUE	NHB OR (95% CI) P-VALUE
Purpura and other hemorrhagic conditions (287)	hematopoietic	63/27/302	2.75 (1.68, 4.51) 5.5e-05	3.74 (1.99, 7.02) 4.25e-05	1.62 (0.658, 4) 0.294
Thrombocytopenia (287.3)	hematopoietic	51/19/289	3.06 (1.84, 5.1) 1.68e-05	4.64 (2.37, 9.08) 7.62e-06	1.66 (0.67, 4.13) 0.272
Secondary thrombocytopenia (287.32)	hematopoietic	48/NA/NA	18.4 (4.94, 68.3) 1.41e-05		
Diseases of white blood cells (288)	hematopoietic	46/13/248	2.46 (1.65, 3.68) 1.13e-05	3.78 (2.18, 6.57) 2.23e-06	1.51 (0.792, 2.88) 0.211
Decreased white blood cell count (288.1)	hematopoietic	238/44/524	2.14 (1.16, 3.94) 0.0143	5.67 (2.36, 13.6) 0.000101	0.797 (0.292, 2.17) 0.658
Neutropenia (288.11)	hematopoietic	108/30/477	4.18 (1.88, 9.27) 0.000436	8.5 (3.03, 23.8) 4.76e-05	1.42 (0.397, 5.11) 0.588
Elevated white blood cell count (288.2)	hematopoietic	66/22/245	2.4 (1.57, 3.67) 5.68e-05	3.68 (2.02, 6.69) 1.96e-05	1.55 (0.79, 3.03) 0.204
Delirium due to conditions classified elsewhere (290.2)	mental disorders	70/428/21	4.18 (2.05, 8.51) 8.04e-05	1.86 (0.726, 4.76) 0.196	27.2 (4.13, 179) 0.000589
Chronic pain syndrome (355.1)	neurological	356/707/41	2.49 (0.971, 6.4) 0.0575	0.611 (0.0985, 3.79) 0.597	9.2 (2.09, 40.6) 0.00338
Hypertensive heart and/or renal disease (401.2)	circulatory system	27/21/45	3.94 (2.32, 6.69) 3.63e-07	5.11 (2.44, 10.7) 1.53e-05	3.51 (1.49, 8.27) 0.00403
Hypertensive chronic kidney disease (401.22)	circulatory system	34/58/42	4.39 (2.39, 8.04) 1.75e-06	5.22 (2.19, 12.5) 0.000201	4.04 (1.58, 10.3) 0.00356
Ischemic Heart Disease (411)	circulatory system	36/34/40	2.45 (1.68, 3.59) 3.55e-06	2.96 (1.74, 5.03) 5.96e-05	2.55 (1.37, 4.77) 0.0033
Myocardial infarction (411.2)	circulatory system	55/42/90	3.03 (1.79, 5.12) 3.43e-05	3.78 (1.94, 7.38) 9.55e-05	3.16 (1.18, 8.46) 0.0224
Coronary atherosclerosis (411.4)	circulatory system	40/76/17	2.56 (1.7, 3.86) 7.07e-06	2.79 (1.56, 4.98) 0.000526	3.34 (1.73, 6.43) 0.000316
Other forms of chronic heart disease (414)	circulatory system	84/215/24	2.89 (1.66, 5.01) 0.000161	2.57 (1.15, 5.72) 0.021	4.02 (1.77, 9.16) 0.000925
Pulmonary heart disease (415)	circulatory system	29/36/38	3.37 (2.1, 5.41) 5.01e-07	3.98 (2.01, 7.89) 7.45e-05	2.76 (1.41, 5.42) 0.00306
Chronic pulmonary heart disease (415.2)	circulatory system	44/75/36	3.58 (2.04, 6.3) 9.52e-06	4.99 (2.01, 12.4) 0.000521	3.11 (1.49, 6.48) 0.00246
Cardiac conduction disorders (426)	circulatory system	90/29/253	1.99 (1.38, 2.86) 0.000222	3.04 (1.78, 5.19) 4.72e-05	1.45 (0.806, 2.61) 0.215
Abnormal electrocardiogram [ECG] [EKG] (426.7)	circulatory system	71/24/211	2.1 (1.45, 3.05) 8.14e-05	3.24 (1.87, 5.6) 2.56e-05	1.54 (0.846, 2.79) 0.158
Other cardiac conduction disorders (426.8)	circulatory system	60/43/170	4.72 (2.24, 9.95) 4.47e-05	6.64 (2.56, 17.2) 9.92e-05	2.9 (0.835, 10.1) 0.0937
Cardiac dysrhythmias (427)	circulatory system	125/47/359	1.76 (1.26, 2.45) 0.000874	2.56 (1.58, 4.15) 0.000132	1.27 (0.734, 2.19) 0.395
Tachycardia NOS (427.7)	circulatory system	42/15/124	2.58 (1.7, 3.91) 7.75e-06	3.99 (2.23, 7.14) 3.19e-06	1.97 (1.01, 3.84) 0.0459
Congestive heart failure; nonhypertensive (428)	circulatory system	53/39/81	2.58 (1.66, 4.01) 2.57e-05	3.66 (1.91, 6.99) 8.79e-05	2.3 (1.15, 4.56) 0.0178
Abnormal function study of cardiovascular system (429.2)	circulatory system	106/33/416	1.98 (1.36, 2.9) 0.000406	3.07 (1.78, 5.31) 5.84e-05	1.24 (0.665, 2.32) 0.498
Hypotension (458)	circulatory system	7/5/10	4.22 (2.78, 6.42) 1.49e-11	4.41 (2.49, 7.81) 3.6e-07	4.75 (2.25, 10) 4.38e-05
Iatrogenic hypotension (458.2)	circulatory system	116/432/26	4.71 (1.92, 11.5) 0.000694	2.34 (0.635, 8.64) 0.201	11.8 (2.67, 52.5) 0.00115
Hypotension NOS (458.9)	circulatory system	14/12/20	4.01 (2.59, 6.2) 5.19e-10	4.28 (2.36, 7.75) 1.66e-06	4.04 (1.83, 8.91) 0.000555

Abbreviations: NHB: non-Hispanic Black/African American; NHW: non-Hispanic Whites; OR: odds ratio; CI: confidence interval; n/a: not available

Table S5 cont'd

TRAIT (PHECODE)	CATEGORY	RANK ALL/NHW/NHB	ALL OR (95% CI) P-VALUE	NHW OR (95% CI) P-VALUE	NHB OR (95% CI) P-VALUE
Other disorders of circulatory system (459)	circulatory system	23/59/11	2.99 (2, 4.46) 7.95e-08	2.88 (1.65, 5.03) 0.000202	3.61 (1.94, 6.73) 5.39e-05
Circulatory disease NEC (459.9)	circulatory system	38/130/18	2.63 (1.73, 3.99) 5.81e-06	2.44 (1.35, 4.41) 0.00302	3.21 (1.68, 6.12) 0.000401
Bacterial pneumonia (480.1)	respiratory	172/451/44	2.56 (1.35, 4.87) 0.00403	1.82 (0.695, 4.75) 0.224	5.06 (1.69, 15.1) 0.00375
Chronic airway obstruction (496)	respiratory	50/25/108	2.83 (1.76, 4.54) 1.67e-05	3.8 (2.02, 7.15) 3.34e-05	2.42 (1.08, 5.45) 0.0326
Pulmonary congestion and hypostasis (503)	respiratory	59/145/39	5.77 (2.49, 13.4) 4.36e-05	5.12 (1.67, 15.7) 0.00431	7.72 (1.99, 29.9) 0.00309
Other pulmonary inflammation or edema (505)	respiratory	26/133/7	6.21 (3.09, 12.5) 2.78e-07	4.61 (1.67, 12.7) 0.00309	11.1 (3.68, 33.3) 1.86e-05
Empyema and pneumothorax (506)	respiratory	15/48/9	5.62 (3.23, 9.77) 1.02e-09	4.77 (2.14, 10.7) 0.000135	6.37 (2.64, 15.4) 3.86e-05
Pleurisy; pleural effusion (507)	respiratory	33/72/57	3.64 (2.15, 6.19) 1.7e-06	3.93 (1.84, 8.39) 0.000405	3.18 (1.38, 7.34) 0.00664
Pulmonary collapse; interstitial and compensatory emphysema (508)	respiratory	16/3/114	6.05 (3.36, 10.9) 2.06e-09	9.08 (4.04, 20.4) 8.98e-08	2.91 (1.06, 8.03) 0.0387
Respiratory failure, insufficiency, arrest (509)	respiratory	1/23/1	4.86 (3.3, 7.15) 1.04e-15	3.48 (1.96, 6.19) 2.23e-05	6.09 (3.26, 11.4) 1.5e-08
Respiratory failure (509.1)	respiratory	5/90/2	5.07 (3.27, 7.84) 3.45e-13	3.08 (1.59, 5.95) 0.000853	6.79 (3.31, 13.9) 1.72e-07
Respiratory insufficiency (509.2)	respiratory	2/14/5	7.92 (4.76, 13.2) 1.94e-15	6.12 (2.88, 13) 2.57e-06	6.6 (2.8, 15.5) 1.6e-05
Pulmonary insufficiency or respiratory failure following trauma and surgery (509.3)	respiratory	3/17/3	18.2 (8.79, 37.7) 5.65e-15	18.1 (5.17, 63.3) 5.85e-06	16.6 (5.73, 47.9) 2.2e-07
Dependence on respirator [Ventilator] or supplemental oxygen (509.8)	respiratory	11/74/6	6.68 (3.74, 11.9) 1.36e-10	4.57 (1.96, 10.7) 0.000453	7.99 (3.1, 20.6) 1.73e-05
Renal failure (585)	genitourinary	9/8/16	3.39 (2.36, 4.89) 5.33e-11	3.81 (2.26, 6.41) 4.92e-07	3.07 (1.67, 5.62) 0.000289
Acute renal failure (585.1)	genitourinary	10/9/25	3.78 (2.52, 5.65) 1.02e-10	4.38 (2.46, 7.81) 5.63e-07	3.04 (1.56, 5.91) 0.00104
Chronic renal failure [CKD] (585.3)	genitourinary	18/18/15	3.27 (2.19, 4.88) 7.38e-09	3.71 (2.1, 6.56) 6.24e-06	3.39 (1.76, 6.54) 0.000272
Renal dialysis (585.31)	genitourinary	41/119/66	4.54 (2.34, 8.81) 7.5e-06	5.37 (1.83, 15.7) 0.00216	3.46 (1.32, 9.1) 0.0118

Abbreviations: NHB: non-Hispanic Black/African American; NHW: non-Hispanic Whites; OR: odds ratio; CI: confidence interval; n/a: not available

Table S6 "Deceased vs. Alive" Merged PheWAS Top 50 Traits Using Adjustment 2

TRAIT (PHECODE)	CATEGORY	RANK ALL/NHW/NHB	ALL OR (95% CI) P-VALUE	NHW OR (95% CI) P-VALUE	NHB OR (95% CI) P-VALUE
Septicemia (038)	infectious diseases	10/114/26	4.31 (2.41, 7.72) 9.03e-07	2.73 (1.05, 7.14) 0.0403	4.41 (1.74, 11.2) 0.00172
Staphylococcus infections (041.1)	infectious diseases	215/25/546	2.92 (1.09, 7.84) 0.0335	7.51 (2.1, 26.9) 0.00194	0.602 (0.0299, 12.1) 0.74
Methicillin sensitive Staphylococcus aureus (041.11)	infectious diseases	179/38/NA	3.82 (1.24, 11.7) 0.0193	9.09 (2.1, 39.3) 0.00316	
Streptococcus infection (041.2)	infectious diseases	212/41/398	3.1 (1.1, 8.7) 0.0321	6.88 (1.9, 24.9) 0.00328	1.98 (0.304, 12.9) 0.475
Secondary diabetes mellitus (249)	endocrine/metabolic	92/12/242	4.4 (1.75, 11) 0.00164	6.9 (2.25, 21.2) 0.000739	2.81 (0.511, 15.4) 0.235
Type 2 diabetes with neurological manifestations (250.24)	endocrine/metabolic	146/366/30	2.78 (1.33, 5.82) 0.00663	1.88 (0.65, 5.44) 0.244	6.52 (1.99, 21.4) 0.00199
Diabetes type 2 with peripheral circulatory disorders (250.25)	endocrine/metabolic	15/19/18	7.38 (3.13, 17.4) 4.99e-06	6.21 (2.06, 18.7) 0.00117	12 (2.73, 53) 0.00102
Disorders of mineral metabolism (275)	endocrine/metabolic	46/284/19	2.8 (1.6, 4.91) 0.000301	1.84 (0.781, 4.33) 0.163	4.21 (1.77, 9.99) 0.00112
Disorders of phosphorus metabolism (275.53)	endocrine/metabolic	32/78/97	4.06 (1.95, 8.47) 0.000183	4.13 (1.29, 13.2) 0.0171	3.26 (1.09, 9.73) 0.034
Disorders of fluid, electrolyte, and acid-base balance (276)	endocrine/metabolic	13/104/8	3.28 (1.99, 5.41) 3.24e-06	2.29 (1.08, 4.87) 0.0306	5.98 (2.38, 15.1) 0.000148
Electrolyte imbalance (276.1)	endocrine/metabolic	16/64/12	3.36 (1.99, 5.66) 5.45e-06	2.84 (1.3, 6.2) 0.00879	5.41 (2.08, 14.1) 0.000555
Hyperosmolality and/or hypernatremia (276.11)	endocrine/metabolic	4/26/3	11.5 (4.86, 27.1) 2.56e-08	11.3 (2.43, 52.6) 0.00198	19.1 (4.56, 79.7) 5.42e-05
Hyposmolality and/or hyponatremia (276.12)	endocrine/metabolic	28/13/93	4.03 (1.99, 8.14) 0.000106	4.95 (1.94, 12.6) 0.000804	3.89 (1.12, 13.5) 0.0322
Hyperpotassemia (276.13)	endocrine/metabolic	39/116/31	3.4 (1.77, 6.51) 0.000226	2.68 (1.04, 6.85) 0.0404	5.68 (1.86, 17.4) 0.00229
Hypopotassemia (276.14)	endocrine/metabolic	21/127/32	4.63 (2.25, 9.55) 3.28e-05	3.07 (1.01, 9.31) 0.0473	5.96 (1.88, 18.9) 0.00239
Acid-base balance disorder (276.4)	endocrine/metabolic	2/45/7	7.05 (3.63, 13.7) 8.14e-09	4.33 (1.58, 11.9) 0.00445	10.6 (3.29, 34.2) 7.79e-05
Acidosis (276.41)	endocrine/metabolic	7/67/11	6.79 (3.38, 13.6) 7.38e-08	4.02 (1.41, 11.5) 0.00948	8.71 (2.55, 29.7) 0.000548
Alkalosis (276.42)	endocrine/metabolic	20/NA/NA	19.1 (4.77, 76.9) 3.17e-05		
Hypovolemia (276.5)	endocrine/metabolic	84/399/40	3.02 (1.55, 5.88) 0.00119	1.68 (0.657, 4.31) 0.278	5.43 (1.78, 16.6) 0.00299
Fluid overload (276.6)	endocrine/metabolic	26/93/67	5.87 (2.44, 14.1) 7.91e-05	4.21 (1.21, 14.7) 0.0243	5.58 (1.45, 21.4) 0.0123
Aplastic anemia (284)	hematopoietic	173/15/NA	5.33 (1.35, 21.1) 0.0172	12.5 (2.78, 55.8) 0.000976	
Pancytopenia (284.1)	hematopoietic	174/16/NA	5.33 (1.35, 21.1) 0.0172	12.5 (2.78, 55.8) 0.000976	
Encounter for long-term (current) use of anticoagulants (286.2)	hematopoietic	190/701/47	2.32 (1.12, 4.79) 0.023	1.29 (0.406, 4.07) 0.669	4.55 (1.57, 13.2) 0.00517
Diseases of white blood cells (288)	hematopoietic	45/2/126	2.86 (1.62, 5.06) 0.000299	4.96 (2.19, 11.3) 0.000128	2.26 (0.938, 5.45) 0.069
Decreased white blood cell count (288.1)	hematopoietic	560/28/612	1.58 (0.605, 4.15) 0.348	8.38 (2.17, 32.4) 0.00206	0.869 (0.197, 3.84) 0.854
Elevated white blood cell count (288.2)	hematopoietic	27/1/119	3.19 (1.78, 5.73) 0.000101	5.86 (2.54, 13.6) 3.51e-05	2.39 (0.951, 5.99) 0.0638

Abbreviations: NHB: non-Hispanic Black/African American; NHW: non-Hispanic Whites; OR: odds ratio; CI: confidence interval; n/a: not available

Table S6 cont'd

TRAIT (PHECODE)	CATEGORY	RANK ALL/NHW/NHB	ALL OR (95% CI) P-VALUE	NHW OR (95% CI) P-VALUE	NHB OR (95% CI) P-VALUE
Delirium dementia and amnestic and other cognitive disorders (290)	mental disorders	25/73/10	4.48 (2.15, 9.34) 6.21e-05	3.53 (1.28, 9.75) 0.0151	8.69 (2.65, 28.5) 0.00036
Delirium due to conditions classified elsewhere (290.2)	mental disorders	11/69/6	8.55 (3.63, 20.2) 9.35e-07	4.55 (1.4, 14.8) 0.0117	15.3 (3.99, 58.4) 6.93e-05
Other persistent mental disorders due to conditions classified elsewhere (290.3)	mental disorders	43/58/64	4.65 (2.03, 10.6) 0.000283	4.72 (1.52, 14.7) 0.0074	6.59 (1.56, 27.9) 0.0104
Alteration of consciousness (291.8)	mental disorders	100/131/49	3.24 (1.54, 6.82) 0.00199	2.87 (1, 8.22) 0.0493	4.97 (1.61, 15.4) 0.00533
Neurological disorders (292)	mental disorders	83/8/152	2.52 (1.44, 4.4) 0.00117	4.41 (1.93, 10.1) 0.000438	2.17 (0.869, 5.42) 0.097
Aphasia/speech disturbance (292.1)	mental disorders	72/6/471	4.44 (1.86, 10.6) 0.000806	7.05 (2.51, 19.8) 0.000212	1.69 (0.225, 12.6) 0.611
Aphasia (292.11)	mental disorders	62/9/NA	8.47 (2.53, 28.4) 0.000534	11.2 (2.9, 43.1) 0.000457	
Mild cognitive impairment (292.2)	mental disorders	91/21/NA	5.79 (1.95, 17.2) 0.00158	9.21 (2.38, 35.7) 0.00132	
Memory loss (292.3)	mental disorders	54/18/361	4.06 (1.87, 8.83) 0.000397	5.2 (1.94, 14) 0.00107	2.03 (0.399, 10.3) 0.394
Altered mental status (292.4)	mental disorders	85/35/52	2.83 (1.5, 5.32) 0.00128	4.02 (1.61, 10) 0.00285	3.82 (1.45, 10.1) 0.00655
Alcoholic liver damage (317.11)	mental disorders	36/NA/NA	13.7 (3.45, 54.5) 0.000202		
Epilepsy, recurrent seizures, convulsions (345)	neurological	263/605/42	2.26 (0.962, 5.33) 0.0614	1.48 (0.403, 5.44) 0.555	7.24 (1.95, 26.9) 0.00311
Convulsions (345.3)	neurological	274/679/41	2.33 (0.942, 5.76) 0.0673	1.45 (0.323, 6.49) 0.629	7.39 (1.97, 27.7) 0.00302
Other conditions of brain (348)	neurological	74/137/20	3.27 (1.63, 6.58) 0.000868	2.68 (0.985, 7.3) 0.0536	5.83 (2.02, 16.9) 0.00113
Retinal vascular changes and abnomalities (362.4)	sense organs	241/36/NA	3.13 (1.02, 9.59) 0.046	9.59 (2.17, 42.4) 0.00287	
Blindness and low vision (367.9)	sense organs	159/34/584	3.31 (1.3, 8.42) 0.0119	6.31 (1.9, 21) 0.00266	1.27 (0.176, 9.19) 0.811
Pulmonary heart disease (415)	circulatory system	40/52/101	3.33 (1.75, 6.33) 0.000237	3.75 (1.46, 9.65) 0.00612	2.63 (1.05, 6.56) 0.0385
Cardiac conduction disorders (426)	circulatory system	77/166/45	2.62 (1.48, 4.64) 0.000943	2.16 (0.932, 4.99) 0.0727	4.5 (1.63, 12.4) 0.00371
Atrioventricular [AV] block (426.2)	circulatory system	47/180/39	5.04 (2.1, 12.1) 0.000302	3.23 (0.857, 12.2) 0.0832	7.49 (2, 28.1) 0.00284
First degree AV block (426.21)	circulatory system	24/149/15	7.25 (2.8, 18.8) 4.54e-05	3.71 (0.946, 14.5) 0.06	122 (7.53, 1980) 0.000723
Abnormal electrocardiogram [ECG] [EKG] (426.7)	circulatory system	68/143/48	2.71 (1.52, 4.85) 0.000739	2.27 (0.973, 5.3) 0.058	4.31 (1.55, 12) 0.00518
Other cardiac conduction disorders (426.8)	circulatory system	107/49/466	5.15 (1.79, 14.9) 0.0024	6.32 (1.75, 22.9) 0.00498	1.77 (0.204, 15.4) 0.604
Cardiac defibrillator in situ (426.92)	circulatory system	150/NA/43	4.55 (1.49, 14) 0.00795		7.84 (1.97, 31.2) 0.00351
Cardiac dysrhythmias (427)	circulatory system	115/328/37	2.29 (1.33, 3.93) 0.00274	1.69 (0.739, 3.87) 0.214	4.64 (1.71, 12.6) 0.00258
Atrial fibrillation and flutter (427.2)	circulatory system	57/367/5	3.4 (1.72, 6.74) 0.00045	1.82 (0.663, 4.99) 0.245	9.42 (3.14, 28.3) 6.31e-05
Atrial fibrillation (427.21)	circulatory system	50/331/4	3.49 (1.76, 6.93) 0.000351	1.9 (0.689, 5.23) 0.215	9.6 (3.2, 28.8) 5.56e-05
Cardiac arrest and ventricular fibrillation (427.4)	circulatory system	38/NA/33	13.6 (3.41, 54.5) 0.00022		16.1 (2.67, 96.5) 0.0024

Abbreviations: NHB: non-Hispanic Black/African American; NHW: non-Hispanic Whites; OR: odds ratio; CI: confidence interval; n/a: not available

Table S6 cont'd

TRAIT (PHECODE)	CATEGORY	RANK ALL/NHW/NHB	ALL OR (95% CI) P-VALUE	NHW OR (95% CI) P-VALUE	NHB OR (95% CI) P-VALUE
Tachycardia NOS (427.7)	circulatory system	65/63/44	3.17 (1.64, 6.12) 0.000585	3.46 (1.37, 8.73) 0.0086	5.65 (1.76, 18.1) 0.00355
Congestive heart failure; nonhypertensive (428)	circulatory system	23/146/27	3.62 (1.95, 6.71) 4.37e-05	2.5 (0.967, 6.46) 0.0587	4.25 (1.71, 10.5) 0.00182
Congestive heart failure (CHF) NOS (428.1)	circulatory system	59/184/50	3.23 (1.68, 6.23) 0.000456	2.36 (0.889, 6.29) 0.0846	3.97 (1.5, 10.5) 0.00557
Heart failure NOS (428.2)	circulatory system	35/294/36	3.37 (1.78, 6.39) 0.000192	2.01 (0.738, 5.5) 0.172	4.24 (1.66, 10.8) 0.00252
Heart failure with reduced EF [Systolic or combined heart failure] (428.3)	circulatory system	48/102/79	3.93 (1.86, 8.31) 0.000346	3.33 (1.12, 9.86) 0.0299	4.04 (1.26, 12.9) 0.0187
Ill-defined descriptions and complications of heart disease (429)	circulatory system	44/97/207	2.71 (1.58, 4.65) 0.000283	2.52 (1.12, 5.7) 0.0262	1.84 (0.766, 4.42) 0.173
Peripheral vascular disease (443)	circulatory system	67/42/394	3.33 (1.66, 6.68) 0.00071	3.62 (1.53, 8.57) 0.00342	1.77 (0.374, 8.4) 0.471
Other specified peripheral vascular diseases (443.8)	circulatory system	41/40/301	3.9 (1.87, 8.13) 0.000275	3.92 (1.58, 9.74) 0.00324	2.25 (0.456, 11.1) 0.319
Peripheral vascular disease, unspecified (443.9)	circulatory system	29/33/332	4.19 (1.98, 8.85) 0.000176	4.12 (1.64, 10.3) 0.00251	2.13 (0.426, 10.7) 0.357
Noninfectious disorders of lymphatic channels (450)	circulatory system	184/47/162	2.92 (1.18, 7.26) 0.0209	6.81 (1.81, 25.6) 0.00448	2.89 (0.769, 10.9) 0.116
Hypotension (458)	circulatory system	42/219/28	2.99 (1.66, 5.41) 0.000279	2.07 (0.844, 5.06) 0.112	4.83 (1.79, 13) 0.00186
Hypotension NOS (458.9)	circulatory system	58/227/35	3.03 (1.63, 5.64) 0.000454	2.1 (0.83, 5.32) 0.117	5.34 (1.8, 15.8) 0.00251
Pulmonary congestion and hypostasis (503)	respiratory	145/860/13	4.36 (1.51, 12.6) 0.00661	1.15 (0.174, 7.57) 0.887	15.3 (3.22, 72.4) 0.000594
Other pulmonary inflamation or edema (505)	respiratory	19/23/23	6.63 (2.81, 15.7) 1.61e-05	7.26 (2.15, 24.6) 0.00144	9.08 (2.34, 35.2) 0.00141
Empyema and pneumothorax (506)	respiratory	30/781/2	4.14 (1.97, 8.7) 0.000178	0.794 (0.131, 4.82) 0.802	10.8 (3.47, 33.6) 3.99e-05
Pleurisy; pleural effusion (507)	respiratory	56/186/34	3.74 (1.79, 7.81) 0.000437	2.65 (0.873, 8.02) 0.0855	6.09 (1.9, 19.6) 0.0024
Respiratory failure, insufficiency, arrest (509)	respiratory	9/115/9	4.07 (2.36, 7.02) 4.76e-07	2.47 (1.04, 5.85) 0.0403	5.67 (2.27, 14.2) 0.000204
Respiratory failure (509.1)	respiratory	17/260/14	4 (2.19, 7.31) 6.53e-06	2.09 (0.781, 5.57) 0.143	5.52 (2.06, 14.8) 0.000673
Respiratory insufficiency (509.2)	respiratory	5/65/17	6.02 (3.2, 11.3) 2.65e-08	3.83 (1.4, 10.5) 0.00914	6.5 (2.16, 19.6) 0.00088
Pulmonary insufficiency or respiratory failure following trauma and surgery (509.3)	respiratory	1/56/1	11 (5.31, 22.9) 1.2e-10	5.45 (1.59, 18.7) 0.00699	15.6 (4.81, 50.5) 4.7e-06
Nephrotic syndrome without mention of glomerulonephritis (580.2)	genitourinary	37/NA/NA	17.9 (3.9, 82.5) 0.000208		
Nephritis and nephropathy with pathological lesion (580.32)	genitourinary	78/44/105	6.3 (2.11, 18.8) 0.000963	9.2 (2.03, 41.8) 0.00404	6.43 (1.09, 38.1) 0.0403
Renal failure (585)	genitourinary	18/59/16	3.45 (2.01, 5.94) 7.55e-06	3 (1.33, 6.74) 0.00799	5.54 (2.02, 15.1) 0.000855
Acute renal failure (585.1)	genitourinary	12/24/29	4.24 (2.37, 7.59) 1.16e-06	3.86 (1.64, 9.05) 0.00192	5.4 (1.87, 15.6) 0.00186
Renal failure NOS (585.2)	genitourinary	97/32/102	5.02 (1.82, 13.9) 0.00181	11.3 (2.38, 53.8) 0.0023	5.41 (1.09, 26.8) 0.039
Chronic renal failure [CKD] (585.3)	genitourinary	22/72/24	3.48 (1.93, 6.29) 3.56e-05	2.9 (1.23, 6.79) 0.0146	5.48 (1.91, 15.7) 0.00153
Renal dialysis (585.31)	genitourinary	34/150/78	6.38 (2.41, 16.9) 0.000191	4.92 (0.929, 26) 0.061	5.97 (1.37, 26) 0.0171

Abbreviations: NHB: non-Hispanic Black/African American; NHW: non-Hispanic Whites; OR: odds ratio; CI: confidence interval; n/a: not available

Table S6 cont'd

TRAIT (PHECODE)	CATEGORY	RANK ALL/NHW/NHB	ALL OR (95% CI) P-VALUE	NHW OR (95% CI) P-VALUE	NHB OR (95% CI) P-VALUE
End stage renal disease (585.32)	genitourinary	31/48/51	5.54 (2.26, 13.6) 0.000179	7.63 (1.87, 31.1) 0.00462	6.22 (1.68, 23.1) 0.00632
Chronic Kidney Disease, Stage IV (585.34)	genitourinary	93/27/114	4.28 (1.73, 10.6) 0.00164	6.96 (2.03, 23.9) 0.00204	4.14 (0.981, 17.5) 0.0532
Chronic kidney disease, Stage I or II (585.4)	genitourinary	120/325/46	3.6 (1.53, 8.47) 0.00331	2.35 (0.617, 8.96) 0.21	6.36 (1.79, 22.6) 0.00419
Kidney replaced by transpant (587)	genitourinary	87/46/167	5.93 (1.99, 17.7) 0.00141	10.1 (2.05, 49.9) 0.00445	3.42 (0.727, 16.1) 0.12
Disorders resulting from impaired renal function (588)	genitourinary	60/31/73	5.03 (2.03, 12.5) 0.000487	8.08 (2.12, 30.7) 0.00217	5.03 (1.38, 18.3) 0.0143
Cellulitis and abscess of arm/hand (681.3)	dermatologic	122/22/243	2.87 (1.41, 5.82) 0.00352	4.74 (1.83, 12.3) 0.0014	2.12 (0.613, 7.35) 0.235
Cellulitis and abscess of leg, except foot (681.5)	dermatologic	73/17/176	3.18 (1.62, 6.28) 0.000822	4.62 (1.85, 11.6) 0.00106	2.4 (0.764, 7.54) 0.134
Cellulitis and abscess of foot, toe (681.6)	dermatologic	33/20/135	3.99 (1.93, 8.26) 0.000186	4.59 (1.82, 11.5) 0.00123	3.36 (0.875, 12.9) 0.0774
Changes in skin texture (687.3)	dermatologic	121/43/NA	7.46 (1.94, 28.7) 0.00348	10.2 (2.1, 49.6) 0.00403	
Disorder of skin and subcutaneous tissue NOS (689)	dermatologic	319/594/38	1.62 (0.898, 2.93) 0.109	1.28 (0.582, 2.82) 0.539	4.58 (1.7, 12.4) 0.00267
Unspecified erythematous condition (695.9)	dermatologic	49/10/NA	8.22 (2.59, 26.1) 0.00035	8.62 (2.5, 29.7) 0.00064	
Osteomyelitis, periostitis, and other infections involving bone (710)	musculoskeletal	152/3/NA	4.65 (1.45, 14.9) 0.00957	20.6 (4.25, 99.9) 0.000172	
Osteomyelitis (710.1)	musculoskeletal	142/4/NA	5.29 (1.61, 17.4) 0.00601	20.6 (4.25, 99.9) 0.000172	
Acute osteomyelitis (710.11)	musculoskeletal	118/7/NA	8.52 (2.09, 34.7) 0.0028	24.8 (4.34, 142) 0.000309	
Unspecified osteomyelitis (710.19)	musculoskeletal	143/5/NA	5.29 (1.61, 17.4) 0.00601	20.6 (4.25, 99.9) 0.000172	
Bursitis (726.3)	musculoskeletal	300/30/431	2.26 (0.869, 5.88) 0.0944	6.11 (1.92, 19.4) 0.00214	0.381 (0.0179, 8.12) 0.536
Musculoskeletal symptoms referable to limbs (771)	symptoms	119/11/232	2.54 (1.38, 4.67) 0.0028	4.27 (1.84, 9.91) 0.000735	1.92 (0.689, 5.36) 0.212
Swelling of limb (771.1)	symptoms	141/50/137	2.25 (1.26, 4.01) 0.006	3.45 (1.45, 8.23) 0.00517	2.19 (0.914, 5.27) 0.0787
Nonspecific findings on examination of blood (790)	symptoms	69/37/230	3.22 (1.63, 6.36) 0.00075	4.35 (1.64, 11.6) 0.00314	2.01 (0.679, 5.96) 0.207
Complications of cardiac/vascular device, implant, and graft (854)	injuries & poisonings	205/39/625	2.77 (1.12, 6.84) 0.0276	5.42 (1.76, 16.7) 0.0032	0.859 (0.141, 5.25) 0.869
Complication due to other implant and internal device (859)	injuries & poisonings	148/29/283	3.94 (1.45, 10.7) 0.00711	9.67 (2.27, 41.1) 0.00212	2.31 (0.492, 10.9) 0.289
Open wound of foot except toe(s) alone (871.3)	injuries & poisonings	14/NA/NA	19.3 (5.48, 68) 4.07e-06		
Hormones and synthetic substitutes causing adverse effects in therapeutic use (962.3)	injuries & poisonings	53/14/180	8 (2.53, 25.3) 0.000394	16.4 (3.13, 85.7) 0.000923	3.98 (0.642, 24.6) 0.138
Sepsis and SIRS (994)	injuries & poisonings	6/76/25	5.21 (2.88, 9.43) 4.78e-08	3.31 (1.25, 8.75) 0.0159	4.69 (1.8, 12.2) 0.00158
Sepsis (994.2)	injuries & poisonings	8/101/22	5.17 (2.83, 9.41) 8.24e-08	3.08 (1.12, 8.41) 0.0287	4.86 (1.85, 12.8) 0.00135
Septic shock (994.21)	injuries & poisonings	3/51/21	11.8 (5.06, 27.3) 1.02e-08	6.56 (1.72, 25) 0.00594	9.6 (2.46, 37.5) 0.00114

Abbreviations: NHB: non-Hispanic Black/African American; NHW: non-Hispanic Whites; OR: odds ratio; CI: confidence interval; n/a: not available
